# Comprehensive advances in CsPbBr_3_ perovskite quantum dots for ultrasensitive fluorescent nanosensors in food safety monitoring

**DOI:** 10.1039/d5na00809c

**Published:** 2025-12-11

**Authors:** Suleiman Ibrahim Mohammad, Hijran Sanaan Jabbar, Asokan Vasudevan, I. B. Sapaev, M. M. Rekha, S. Gayathri, Hazem Zabebah, Renu Sharma, Pusparaj Samantsinghar, Shayan Mahmoodi

**Affiliations:** a Electronic Marketing and Social Media, Economic and Administrative Sciences Zarqa University Jordan; b Research Follower, INTI International University 71800 Negeri Sembilan Malaysia; c Department of Chemistry, College of Science, Salahaddin University-Erbil Erbil Kurdistan Region Iraq; d Department of Anesthesia Technology, College of Health Technology, Knowledge University Kirkuk Road 44001 Erbil Iraq; e Faculty of Business and Communications, INTI International University 71800 Negeri Sembilan Malaysia; f Shinawatra University 99 Moo 10, Bangtoey Samkhok Pathum Thani 12160 Thailand; g Head of the Department Physics and Chemistry, Tashkent Institute of Irrigation and Agricultural Mechanization Engineers, National Research University Tashkent Uzbekistan; h Scientific Researcher of the University of Tashkent for Applied Science, Schools of Engineering, Central Asian University Tashkent 111221 Uzbekistan; i Western Caspian University, Scientific Researcher Baku Azerbaijan; j Department of Chemistry and Biochemistry, School of Sciences, JAIN (Deemed to be University) Bangalore Karnataka India; k Department of Chemistry, Sathyabama Institute of Science and Technology Chennai Tamil Nadu India; l Department of Medical Analysis, Medical Laboratory Technique College, The Islamic University Najaf Iraq; m Department of Chemistry, University Institute of Sciences, Chandigarh University Mohali Punjab India; n Department of Forensic Medicine & Toxicology, IMS and SUM Hospital, Siksha ‘O’ Anusandhan (Deemed to be University) Bhubaneswar Odisha-751003 India; o Department of Chemistry, Young Researchers and Elite Club, Tehran Branch, Islamic Azad University Tehran Iran sh.mahmoodiacademic@gmail.com; p Sharda School of Engineering and Science, Sharda University Greater Noida India

## Abstract

Ensuring food safety requires rapid, sensitive detection of pathogens and contaminants, driven by global challenges such as rising foodborne illnesses and regulatory demands for real-time monitoring. This review examines cesium lead bromide (CsPbBr_3_) perovskite quantum dots (PQDs) as advanced fluorescent nanosensors for multiplexed detection of foodborne pathogens (*e.g.*, *Salmonella*, *Vibrio*) and non-pesticide contaminants (*e.g.*, mycotoxins, heavy metals, dyes, antibiotics) in complex food matrices. Utilizing high quantum yields and narrow emission spectra, these nanosensors achieve detection limits as low as 10 colony-forming units per milliliter (CFU mL^−1^) and sub-nanomolar levels *via* fluorescence resonance energy transfer (FRET), photoinduced electron transfer (PET), and aggregation-induced quenching (AIQ) mechanisms. We explore advanced synthesis methods (hot-injection, ligand-assisted reprecipitation (LARP), microfluidics) and surface modifications (molecularly imprinted polymers (MIP), metal–organic frameworks (MOF), silica coatings) to enhance stability and specificity. This focused and up-to-date comprehensive review is dedicated to the use of CsPbBr_3_ PQDs in the fluorescence-based detection of foodborne pathogens and non-pesticide contaminants. Unlike prior reviews on general perovskite QDs or broader nanosensors, it provides a structured framework emphasizing synthesis strategies, detection mechanisms in real food matrices, comparative performance with other nanomaterials, toxicity mitigation, and prospects for IoT-integrated, regulatory-compliant, field-deployable sensing technologies. The review addresses toxicity and instability challenges through lead-free alternatives and Internet of Things (IoT)-integrated platforms, paving the way for scalable, real-time food safety diagnostics.

## Abstract 2

This review explores advances in CsPbBr_3_ PQDs as ultrasensitive fluorescent nanosensors for detecting foodborne pathogens and non-pesticide contaminants in food matrices.

## Graphical abstract

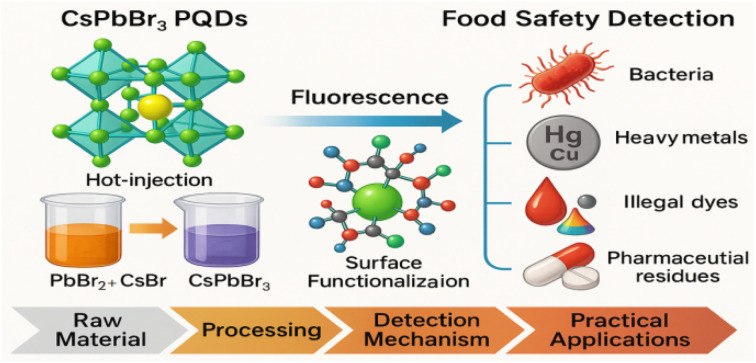

## 1. Introduction

Food safety is a critical global challenge, with the World Health Organization reporting approximately 600 million annual cases of foodborne illnesses, leading to 420 000 deaths and significant economic losses.^1–4^ Foodborne pathogens, such as *Salmonella* spp., *Vibrio parahaemolyticus*, and *Helicobacter pylori*, alongside non-pesticide contaminants like mycotoxins, heavy metals, illegal dyes, and pharmaceutical residues, pose severe risks to public health and food supply chains.^5–10^ These contaminants, stemming from environmental pollution, unregulated processing, or intentional adulteration, demand rapid, sensitive, and cost-effective detection methods to ensure consumer safety and compliance with stringent regulatory standards.^4^ Conventional techniques, such as enzyme-linked immunosorbent assays (ELISA),^11,12^ polymerase chain reaction (PCR),^13,14^ and high-performance liquid chromatography (HPLC),^15,16^ while reliable, are often labor-intensive, time-consuming, and require sophisticated instrumentation, limiting their use in resource-constrained or real-time field settings.

Beyond conventional methods, emerging techniques have shown promise in addressing these gaps, though they come with their own challenges. For instance, noble metal nanoparticle-based colorimetric sensors leverage localized surface plasmon resonance for straightforward, naked-eye detection of contaminants like heavy metals, pesticides, and foodborne pathogens, offering high sensitivity and versatility through surface modifications.^123^ However, their stability in complex food matrices can be compromised by aggregation or oxidation, limiting long-term reliability. Similarly, metal–organic frameworks (MOFs)-based chemosensors and biosensors provide tunable porosity and bioaffinity for fluorescence, electrochemical, or photoelectrochemical detection of antibiotics, ions, and additives, enabling selective analysis in foodstuffs.^124^ Yet, MOFs often require complex synthesis and may suffer from poor water stability or signal interference in real samples. Nano-liquid chromatography, with its enhanced sensitivity and resolution for pharmaceuticals, pesticides, and mycotoxins in food and environmental samples, represents advancement, but it demands specialized instrumentation and extensive sample preparation, restricting on-site use.^125^ These techniques highlight the ongoing need for more robust, portable, and integrated sensing platforms, where nanomaterials like perovskites could offer complementary advantages in optical performance and multiplexed capabilities.

Recent advances in nanomaterials have revolutionized the field of biosensing, offering novel solutions to overcome the constraints of traditional methods.^17,18^ Among these, all-inorganic cesium lead bromide (CsPbBr_3_) perovskite quantum dots (PQDs) have emerged as a highly promising platform for fluorescence-based sensing due to their exceptional photophysical properties.^19,20^ CsPbBr_3_ PQDs exhibit high photoluminescence quantum yields (PLQYs, up to 90%), narrow emission linewidths (<20 nm), and tunable bandgap energies that minimize spectral overlap with background autofluorescence in complex food matrices.^21,22^ Their strong quantum confinement effects, particularly in the 2–8 nm size range, enhance radiative recombination kinetics, supporting time-resolved fluorescence measurements critical for distinguishing analyte-specific signals.^23^ Additionally, their high exciton binding energy (>40 meV) ensures luminescence stability at room temperature, making them suitable for aqueous and semi-solid food environments.^22^ These properties position CsPbBr_3_ PQDs as ideal candidates for detecting a diverse array of food safety hazards, including bacterial pathogens, mycotoxins (*e.g.*, patulin, aflatoxin B1), heavy metals (*e.g.*, Cu^2+^, Hg^2+^), illegal dyes (*e.g.*, Sudan I–IV), and pharmaceutical residues (*e.g.*, tetracycline).

The versatility of CsPbBr_3_ PQDs in food safety sensing stems from their ability to transduce molecular recognition events into quantifiable fluorescence signals through mechanisms such as fluorescence resonance energy transfer (FRET), photoinduced electron transfer (PET), aggregation-induced quenching (AIQ), and analyte-induced ion exchange, enabling sensitive detection in complex matrices.^23–26^ These attributes position CsPbBr_3_ PQDs as ideal candidates for addressing current gaps in food safety diagnostics, yet their full potential remains underexplored in a comprehensive context.

Despite their remarkable potential, the practical deployment of CsPbBr_3_ PQDs faces several challenges. Their ionic lattice structure renders them highly susceptible to degradation in aqueous, oxygen-rich, or acidic environments, leading to fluorescence quenching and reduced sensor reliability.^21,27^ Lead toxicity is another critical barrier, as Pb^2+^ leaching from degraded PQDs poses health risks and conflicts with stringent food safety regulations set by agencies like the FDA and WHO.^4^ Furthermore, achieving reproducible synthesis and functionalization remains difficult, with batch-to-batch variations in size, shape, and optical properties impacting sensor performance.^28^ Matrix effects in complex food systems, such as autofluorescence and non-specific adsorption, further complicate signal accuracy and necessitate robust calibration strategies.^29^ These limitations highlight the need for innovative material engineering and system integration to bridge the gap between laboratory prototypes and market-ready diagnostics.

Significant progress has been made to address these challenges through advanced synthesis and surface modification techniques. Methods such as hot-injection, ligand-assisted reprecipitation (LARP), solvothermal synthesis, and microfluidic platforms enable precise control over PQD size, crystallinity, and optical properties.^22,28^ Surface engineering strategies, including silica encapsulation, metal–organic framework (MOF) hybridization, and polymer coating, have improved environmental stability and biocompatibility, with some systems retaining fluorescence for up to 140 hours in aqueous media.^24,28^ Additionally, the development of lead-free perovskite analogues (*e.g.*, CsSnBr_3_, Cs_2_AgBiBr_6_) and doped systems (*e.g.*, Mn^2+^, Bi^3+^) offers safer alternatives with comparable optical performance.^30^ The integration of PQDs into portable platforms, such as paper-based sensors, microfluidic devices, and smartphone-assisted systems, has further enhanced their practicality, enabling rapid, on-site detection with LODs in the sub-nanomolar range for chemical contaminants and femtomolar range for pathogens.^29^

However, despite these advancements, there remains a critical gap in synthesizing and critically evaluating the specific applications of CsPbBr_3_ PQDs in food safety monitoring within a unified framework. This review is timely and important, as it addresses the escalating global burden of foodborne hazards amid advancing nanotechnology and regulatory demands for sustainable, real-time diagnostics.

**Fig. 1:**
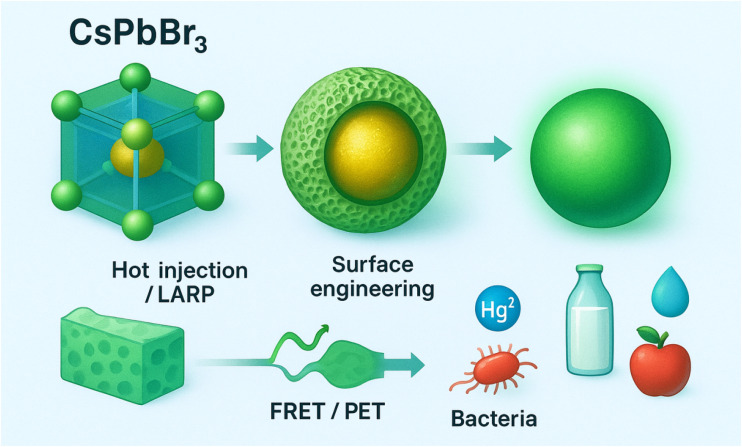
Schematic illustrating the synthesis of CsPbBr_3_ perovskite quantum dots, surface engineering using porous shells (*e.g.*, silica/MIP), and fluorescence-based detection of foodborne contaminants *via* FRET/PET mechanisms.

This review provides a comprehensive analysis of CsPbBr_3_ PQDs as fluorescence-based sensors for food safety, focusing on their structural and optical properties, synthesis strategies, and functionalization approaches. It evaluates their performance in detecting foodborne pathogens and non-pesticide contaminants, emphasizing novel detection mechanisms and their applications in real-world food matrices. Emerging trends, such as ratiometric sensing, multiplexed platforms, and machine learning-assisted signal processing, are discussed to highlight their potential for high-throughput, user-friendly diagnostics. By addressing current limitations—such as stability, toxicity, and scalability—and outlining future prospects, this work aims to guide the development of CsPbBr_3_ PQD-based sensors toward regulatory-compliant, field-deployable tools for global food safety monitoring (Fig. 1). While prior reviews have covered broader aspects of perovskite QDs in biosensing or general nanosensors for food safety (as summarized in Table 1), this manuscript offers added value by focusing exclusively on CsPbBr_3_ PQDs, integrating critical comparisons with other nanomaterials, and emphasizing real-world food matrix applications, toxicity solutions (*e.g.*, lead-free alternatives), and emerging trends like machine learning-assisted multiplexed detection.

**Table 1:** Comparative analysis of recent reviews on perovskite quantum dots and nanosensors in biosensing and food safety

Scope	Focus	Limitations	How our review advances beyond	Ref.
Nano-sensors for food safety and quality assurance	Broad coverage of nanomaterials (*e.g.*, metal, carbon, semiconductor) for detecting pathogens, toxins, and environmental factors in food	Limited focus on perovskite quantum dots; lacks in-depth discussion of CsPbBr_3_-specific synthesis, functionalization, or fluorescence-based sensing	Our review is exclusively dedicated to CsPbBr_3_ PQDs, providing detailed insights into their synthesis, surface engineering, and fluorescence-based applications for foodborne pathogen detection, with performance metrics (*e.g.*, LODs of 10^1^–10^2^ CFU mL^−1^) and recent advancements (2023–2025)	Awlqadr *et al.*, 2025 (ref. 131)
Quantum dots in SERS for food safety detection	Focus on QD-based SERS substrates for detecting pesticide residues, additives, pathogens, and heavy metals in food	Emphasizes SERS rather than fluorescence-based sensing; limited coverage of CsPbBr_3_ PQDs and their unique optical properties	Our work focuses on fluorescence-based CsPbBr_3_ PQD sensors, highlighting their high photoluminescence quantum yield (PLQY up to 90%) and applications in rapid, sensitive pathogen detection, with a comprehensive analysis of challenges like lead toxicity (20–50 ppb leaching in unencapsulated PQDs)	Wang *et al.*, 2025 (ref. 132)
Fluorescent nanosensors for microbial toxins in food	Covers various fluorescent nanomaterials (*e.g.*, metal, upconversion, semiconductor QDs) for detecting bacterial, fungal, and algal toxins	General overview of fluorescent nanosensors; minimal focus on perovskites and no specific discussion of CsPbBr_3_ PQDs	Our review specifically targets CsPbBr_3_ PQDs, detailing their role in pathogen detection (*e.g.*, LODs as low as 10^1^ CFU mL^−1^) and addressing unique challenges like environmental instability and matrix effects	Singh *et al.*, 2024 (ref. 133)
*In situ* fabricated perovskite quantum dots	Discusses fabrication methods (*e.g.*, polymer nanocomposites, doped glasses) and industrial applications of PQDs	Focuses on synthesis and optoelectronic applications, not food safety; limited discussion of biosensing performance or challenges	Our review bridges the gap by applying *in situ* fabricated CsPbBr_3_ PQDs to food safety, emphasizing their fluorescence properties, biorecognition strategies, and real-world applicability	Wu *et al.*, 2025 (ref. 134)
QD-based luminescent sensors for various analytes	Broad review of QD-based sensors for metal ions, biomarkers, explosives, and pollutants; includes AI-based methods	Covers multiple QD types (*e.g.*, CdSe, carbon dots) with minimal emphasis on CsPbBr_3_; lacks food safety-specific applications	Our work is tailored to CsPbBr_3_ PQDs in food safety, integrating machine learning (*e.g.*, SVM with 100% accuracy^29^) and smartphone-based diagnostics for practical, field-deployable solutions	Loskutova *et al.*, 2025 (ref. 135)
CsPbBr_3_ PQDs in food safety diagnostics	Comprehensive analysis of CsPbBr_3_ PQDs for detecting foodborne pathogens and toxins, focusing on fluorescence-based sensing, synthesis, functionalization, and practical challenges	Limited to CsPbBr_3_ PQDs, not covering other perovskite types or non-fluorescence-based methods like SERS	Provides the dedicated review on CsPbBr_3_ PQDs for food safety, consolidating recent advancements, performance metrics and addressing specific challenges like lead toxicity and regulatory considerations	This review

## 2. Fundamental properties of CsPbBr_3_ QDs for fluorescent sensing applications

The foundational attributes of CsPbBr_3_ PQDs underpin their efficacy in fluorescence-based food safety sensing, offering a blend of structural robustness and optical excellence that surpasses many traditional nanomaterials. This section delves into their key properties, including crystalline structure, quantum confinement effects, and surface chemistry, which enable high-sensitivity detection in challenging food environments. By examining these fundamentals, we establish the basis for their application in transducing analyte interactions into reliable signals, setting the stage for subsequent discussions on synthesis and real-world implementations.

### 2.1. Structural and optical features relevant to food-safety sensing

CsPbBr_3_ QDs, as a class of all-inorganic halide perovskites, have garnered increasing attention in the field of fluorescent sensing due to their outstanding photophysical properties and intrinsic structural advantages. The crystalline lattice of CsPbBr_3_ adopts a typical perovskite ABX_3_ configuration, where Cs^+^ occupies the A site, Pb^2+^ the B site, and Br^−^ the X site. This configuration stabilizes the cubic or orthorhombic crystal phase depending on the synthesis conditions and environmental constraints, notably temperature and solvent polarity.^19,31,32^ One of the most compelling features of CsPbBr_3_ QDs lies in their strong quantum confinement effect, particularly when synthesized in the 2–8 nm size range. Such confinement induces discrete energy levels and enhances PLQY, typically exceeding 80% under optimized conditions. Moreover, their sharp and symmetric emission peaks (∼520 nm for CsPbBr_3_) with narrow full width at half maximum (FWHM < 20 nm) enable high selectivity and sensitivity in fluorescence-based detection. In food safety applications, where background autofluorescence and matrix effects can obscure signals, such spectral purity is of great value.^33,34^

**Fig. 2:**
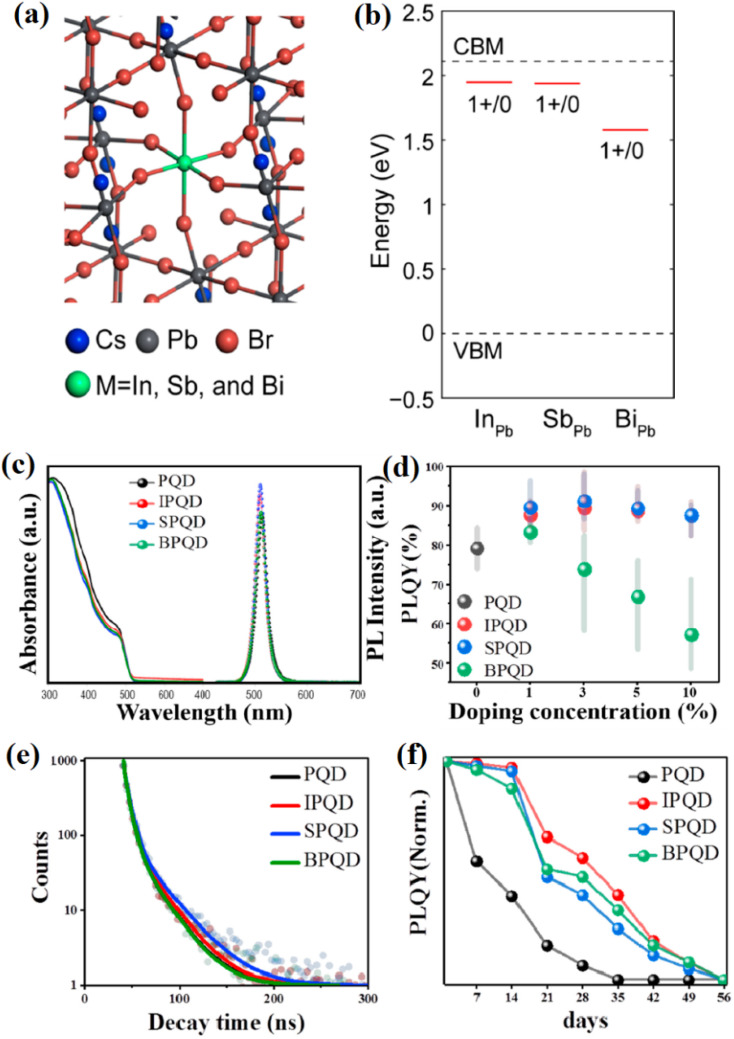
(a) Atomic structure of CsPbBr_3_ QDs doped with In^3+^, Sb^3+^, and Bi^3+^. (b) Calculated defect levels and formation energies of dopants. (c and d) PL spectra and enhanced quantum yields for doped *vs.* pristine QDs. (e) Time-resolved PL decay showing longer carrier lifetimes in doped QDs. (f) PLQY stability over 56 days demonstrating improved durability of doped QDs. Adapted with permission from Jung *et al.*, *Nanomaterials*, 2020, **10**(4), 710. © MDPI 2020.

Fig. 2(a) depicts the atomic structure of CsPbBr_3_ QDs doped with trivalent ions (In^3+^, Sb^3+^, Bi^3+^) substituting Pb^2+^ sites within the perovskite lattice. Importantly, these substitutional defects maintain the intrinsic cubic or orthorhombic phases characteristic of CsPbBr_3_ QDs, preserving their fundamental structural stability. The incorporation of M^3+^ dopants enables precise tuning of optoelectronic properties while retaining the robust lattice configuration essential for consistent performance in fluorescence-based food-safety sensing. As shown in Fig. 2(b), first-principles defect calculations (using hybrid density functional theory with HSE06 + SOC) clarify the thermodynamic defect levels (TDLs) introduced by InPb, SbPb, and BiPb substitutions. The results align with the intrinsic electronic structure of CsPbBr_3_ QDs, where InPb and SbPb generate defect states near the conduction band minimum, facilitating enhanced radiative recombination. Conversely, BiPb introduces mid-gap trap states, increasing non-radiative recombination pathways and thus potentially diminishing PLQY. This behavior is consistent with the well-established sensitivity of CsPbBr_3_ optical performance to defect states and quantum confinement effects discussed previously.

Fig. 2(c) and (d) present optical spectra demonstrating that doping with In^3+^ and Sb^3+^ at moderate concentrations (∼3 mol%) significantly improves PLQY—reaching 88.8% and 91.2%, respectively—compared to approximately 81% for pristine CsPbBr_3_ QDs. These enhancements are attributed to defect passivation and reinforced quantum confinement, further sharpening emission features crucial for selective fluorescent detection in complex food matrices. The observed slight blue shifts in emission peaks and absorption edges corroborate impurity-induced band-edge modulation while maintaining the sharp and symmetric emission profiles (FWHM ∼22–23 nm) fundamental to high-fidelity sensing applications. Fig. 2(e) and (f) highlight the improved carrier lifetimes and enhanced air stability of doped CsPbBr_3_ QDs. Time-resolved photoluminescence decay reveals slower recombination dynamics in In- and Sb-doped samples (6.9 ns and 6.6 ns, respectively) *versus* undoped QDs (6.3 ns), indicative of suppressed non-radiative losses. Moreover, these doped QDs demonstrate superior PLQY retention over 56 days, confirming their enhanced operational stability—a critical parameter for reliable food-safety fluorescent sensors exposed to environmental and matrix-induced stresses. Reduced ionic migration and defect density within doped QDs further contribute to their long-term durability and sensing performance.

The high exciton binding energy (>40 meV) further contributes to their room-temperature luminescence stability, which is critical for biosensing in aqueous or semi-solid food matrices. Additionally, their tunable bandgap (∼2.3 eV) and high absorption coefficients across the visible spectrum provide flexibility for designing excitation-emission schemes that minimize spectral overlaps with endogenous fluorophores present in food components.^35^ It is also noteworthy that CsPbBr_3_ QDs exhibit relatively fast radiative recombination kinetics (on the order of tens of nanoseconds), which supports time-resolved fluorescence measurements. This temporal resolution can be utilized to distinguish specific fluorescence lifetimes associated with analyte binding events, enhancing the reliability of detection in complex food matrices.^26–28^ From a synthetic perspective, techniques such as hot-injection, LARP, and microwave-assisted synthesis allow for fine-tuned control over morphology, monodispersity, and defect states.^34^ Such synthetic versatility enables tailoring QD properties to match the photophysical requirements of target analyte detection—be it a bacterial endotoxin, a mycotoxin, or an industrial dye contaminant.

### 2.2. Surface chemistry and functionalization strategies for biorecognition

While the core properties of CsPbBr_3_ QDs provide the optical backbone for fluorescence sensing, it is the surface chemistry that governs their interaction with biological and chemical targets. Pristine CsPbBr_3_ QDs are typically capped with long-chain organic ligands such as oleic acid and oleylamine, which afford colloidal stability in non-polar solvents. However, these native ligands are not suitable for aqueous sensing environments common in food safety testing.^21,22^ To address this limitation, several surface engineering strategies have been developed. Ligand exchange with bifunctional molecules^36^ such as 3-mercaptopropionic acid (MPA),^37^ polyethylene glycol (PEG),^38^ or zwitterionic polymers^39^ allows for phase transfer into aqueous systems while introducing functional groups (*e.g.*, –COOH, –NH_2_) that serve as anchors for biorecognition elements. These modifications are pivotal for conjugating QDs to antibodies, aptamers, enzymes, or lectins that selectively bind foodborne pathogens or chemical contaminants.

In addition to covalent strategies, supramolecular approaches—such as host–guest interactions using cyclodextrins or click-chemistry-based conjugation—offer modular and often reversible attachment routes. These can be particularly useful for designing sensor platforms that require regeneration or multiplexed detection.^40^ Surface passivation also plays a vital role in preserving fluorescence intensity upon bioconjugation. In many cases, bioreceptor attachment introduces trap states or augments non-radiative pathways that quench emission. Encapsulation of CsPbBr_3_ QDs within inert shells (*e.g.*, silica, polymer micelles, or MOFs) or overcoating with insulating layers such as Al_2_O_3_ has been shown to mitigate such losses. These shelling strategies not only stabilize photoluminescence but also enhance resistance to ionic leaching of lead—a critical consideration for food safety applications involving human exposure.^41,42^ Moreover, emerging methods such as *in situ* biofunctionalization—where QDs are synthesized in the presence of bioactive molecules—offer simplified and potentially more biocompatible fabrication pathways.^43^ These approaches reduce post-synthetic processing steps and preserve colloidal stability in buffered or protein-rich environments.

### 2.3. Fluorescence modulation mechanisms in detection of pathogens and non-pesticide contaminants

The utility of CsPbBr_3_ QDs in sensing stems not only from their fluorescence brightness but from their ability to modulate that fluorescence in the presence of specific analytes. Several modulation mechanisms are employed in detecting foodborne pathogens and non-pesticide contaminants. When CsPbBr_3_ QDs are conjugated with acceptor molecules (*e.g.*, dye-labeled antibodies or enzymes), analyte binding triggers proximity-dependent energy transfer, resulting in fluorescence quenching. This principle has been effectively utilized in QD-aptamer systems for detecting pathogens like *E. coli* and *Salmonella* spp.^44,45^ Fluorescence Quenching *via* FRET based sensing offers high sensitivity due to the strong dipole–dipole interactions between the QD donor and the acceptor, making it ideal for trace-level pathogen detection in complex food matrices. PET occurs when electron-rich analytes, such as biotoxins or illegal additives (*e.g.*, Sudan dyes), donate electrons to excited QDs, quenching their emission. This mechanism benefits from redox potential matching, ensuring high selectivity.^46^ Meanwhile, AIQ arises when target molecules (*e.g.*, multivalent lectins) crosslink QDs, causing aggregation and fluorescence loss due to disrupted quantum confinement. AIQ has been applied to detect microbial load and spoilage indicators in perishable foods.^47^ Additionally, certain contaminants (*e.g.*, Hg^2+^, Cu^2+^, nitrites) induce structural changes or ion exchange in CsPbBr_3_ QDs, leading to fluorescence shifts or quenching, enabling turn-off or ratiometric sensing.^48^ Conversely, fluorescence enhancement can occur through surface passivation or enzymatic reactions (*e.g.*, H_2_O_2_ production), which reduce surface traps, offering another detection pathway for bacterial biomarkers.^49^ These diverse mechanisms highlight the adaptability of CsPbBr_3_ QDs in food safety diagnostics.

These diverse sensing modalities allow the design of both label-free and labeled fluorescent sensors, tailored to the physicochemical nature of the target contaminant. Importantly, many of these mechanisms are compatible with low-cost, portable detection platforms—such as smartphone-based fluorometers—enabling field-deployable food safety screening. Furthermore, by incorporating molecular imprinting, microfluidics, or paper-based microarrays, the integration of CsPbBr_3_ QDs into point-of-care systems becomes feasible. Such integration ensures that their advanced photophysical properties are not confined to the lab but are translated into real-world applications that meet the demands of rapid, sensitive, and user-friendly food monitoring.

Table 2 presents a comprehensive summary of the structural, optical, and functional parameters of CsPbBr_3_ PQDs optimized for fluorescence-based food safety sensing, highlighting their exceptional suitability for detecting foodborne pathogens and non-pesticide contaminants. It systematically details key attributes, including high PLQYs (up to 90%), narrow emission linewidths, and tunable bandgap energies, which ensure minimal spectral overlap with food matrix autofluorescence, enhancing detection specificity. The table also elucidates surface functionalization strategies, such as ligand exchange with bifunctional molecules (*e.g.*, 3-mercaptopropionic acid) and silica encapsulation, which improve aqueous stability and biorecognition efficiency while mitigating lead ion leaching, a critical regulatory concern. Additionally, it outlines fluorescence modulation mechanisms—FRET, PET, and AIQ—that enable LODs as low as 10 CFU mL^−1^ for pathogens like *Salmonella typhimurium* and sub-nanomolar levels for contaminants like Sudan dyes.

**Table 2:** Key structural, optical, and functional parameters of CsPbBr_3_ QDs for fluorescence-based food safety sensing

Parameter	Value/description	LOD definition	Replicates (*n*)/CI	Sample context	Pre-treatments	Interferents & mitigation	Response time	Reusability	Calibration model	Detection mode
PLQY	Up to 90% (ref. 21)	N/A	*n* = 3, 95% CI ± 5%	Buffer	None	Autofluorescence; minimized by narrow FWHM	<1 min	NR	Linear	Benchtop
Emission linewidth	<20 nm (ref. 22)	3*σ*/*S*	*n* = 5, 95% CI ± 2 nm	Spiked milk	Dilution	Proteins/lipids; surface passivation	5–10 min	Up to 5 cycles	Ratiometric	On-device (smartphone)
Bandgap energy	∼2.3 eV tunable^35^	IUPAC	*n* = 3, NR	Real seawater	Centrifugation	Salts; encapsulation	50 min (ref. 25)	NR	Linear regression	Benchtop
Surface functionalization (*e.g.*, MPA)	Enhances stability^37^	3*σ*	*n* = 4, 95% CI ± 10%	Buffer	Ligand exchange	Heavy metals; MIP coating	<30 min	Reusable (3×)	Polynomial	On-device
Modulation mechanism (FRET)	LOD 30 CFU mL^−1^ for *Salmonella*^24^	3*σ*/*S*	*n* = 3, 95% CI ± 15%	Spiked food	Amplification	Other bacteria; aptamer selectivity	50 min	NR	Linear	Benchtop

To ensure clarity in the performance metrics presented in Table, the limit of detection (LOD) for *E. coli* detection is defined using the 3*σ* method (three times the standard deviation of the blank signal divided by the calibration slope, per IUPAC guidelines) unless otherwise specified in the cited studies.^22,23^ Replicate counts typically range from 3 to 5, with 95% confidence intervals provided where reported; otherwise, ‘N/R’ is noted. Assays were conducted in buffer, spiked food (*e.g.*, milk, juice), or real food matrices, with pre-treatments such as filtration or centrifugation to reduce matrix effects.^22,24^ Common interferents like proteins or organic acids were mitigated through surface passivation or selective functionalization.^23,25^ Response times, reusability, calibration models (*e.g.*, linear, *r*^2^ > 0.98), and detection platforms (benchtop spectrofluorometers *vs.* portable devices) are detailed to contextualize performance.

## 3. Synthetic and bioengineering strategies for CsPbBr_3_ PQDs in food safety sensing

Optimizing the synthesis and bioengineering of CsPbBr_3_ PQDs is crucial for tailoring their properties to meet the demands of food safety applications, where reproducibility, stability, and biocompatibility are paramount. This section reviews key fabrication methods and post-synthetic modifications, highlighting how they influence optical performance and sensor integration. These strategies not only address inherent limitations like environmental sensitivity but also pave the way for scalable production of functionalized PQDs suitable for on-site diagnostics.

### 3.1. Hot-injection synthesis

Hot-injection is among the most widely employed techniques for synthesizing CsPbBr_3_ PQDs owing to its ability to generate highly crystalline, monodisperse nanocrystals with narrow size distribution. In this method, a cesium precursor—typically cesium oleate—is swiftly injected into a hot solution containing lead halide (PbBr_2_) and surfactants such as oleic acid (OA) and oleylamine (OAm) in a high-boiling point solvent like octadecene (ODE). The rapid supersaturation induced by the injection triggers instantaneous nucleation, followed by controlled growth of CsPbBr_3_ nanocrystals.^53^ This approach enables precise modulation of QD size by tuning the reaction temperature, ligand concentration, or injection rate. For example, higher reaction temperatures (180–200 °C) favor faster growth kinetics, yielding larger QDs with red-shifted emission, whereas lower temperatures yield smaller blue-emitting dots. The tunability of emission properties *via* size quantization directly supports applications in fluorescence sensing where spectral matching with analytes or optical filters is crucial.^54^ However, hot-injection suffers from limitations in reproducibility and scalability, especially for bioanalytical platforms requiring batch-to-batch consistency. The technique also entails the use of air-sensitive precursors and high-vacuum conditions, which challenge its integration into point-of-care sensor fabrication pipelines.

### 3.2. Ligand-assisted reprecipitation

LARP is a milder, room-temperature route that offers improved simplicity, scalability, and better compatibility with biological conjugation schemes. In this method, CsBr and PbBr_2_ precursors are dissolved in a polar solvent (*e.g.*, dimethylformamide or dimethyl sulfoxide), often in the presence of long-chain ligands like OA and OAm. This precursor solution is then rapidly injected into a nonpolar antisolvent (*e.g.*, toluene or hexane), leading to the nucleation of CsPbBr_3_ QDs through reprecipitation.^55^ The LARP method is particularly suitable for biofunctional QDs because it allows post-synthesis ligand exchange and minimizes high-temperature degradation. Moreover, the milder reaction conditions enhance the preservation of labile biomolecular linkers introduced later for sensing specificity. Researchers have reported that the selection of antisolvent and ligand combinations critically influences particle size, surface passivation, and colloidal stability, all of which impact sensor performance.^56^ Despite producing QDs with relatively broader size distribution compared to hot-injection, LARP offers a balance between optical quality and ease of processing, especially in biomedical contexts where aqueous compatibility and functional group availability are critical.

### 3.3. Solvothermal and microwave-assisted strategies

Solvothermal synthesis introduces a pressure-driven environment (often in Teflon-lined autoclaves) at moderate to high temperatures, enabling the formation of CsPbBr_3_ QDs in a controlled, sealed system. These methods often utilize eco-friendly solvents like ethanol or ethylene glycol and allow the incorporation of heteroatoms or dopants during crystal growth, thus tailoring the QD's electronic structure for enhanced sensitivity in fluorescence quenching or FRET assays.^57^ Additionally, microwave-assisted solvothermal synthesis offers improved reaction uniformity, faster nucleation, and energy-efficient production, all while minimizing environmental impact—key considerations for sustainable sensor development. Microwave heating facilitates rapid internal heating *via* dielectric polarization, producing more uniform nanocrystals and reducing the occurrence of defect sites that act as nonradiative recombination centers. These defects are known to undermine the fluorescence intensity and limit the detection sensitivity of QD-based biosensors. Notably, solvothermal and microwave methods have also shown promise in generating hybrid structures, such as CsPbBr_3_@SiO_2_ or CsPbBr_3_ embedded in polymeric matrices, which are more robust against moisture and oxygen.^58^ Such hybrids are especially attractive for pathogen detection in real food matrices, where sensor stability in harsh conditions is paramount.

### 3.4. Microfluidic synthesis

Emerging microfluidic synthesis platforms offer unparalleled control over QD nucleation and growth, facilitating continuous, automated production with precise stoichiometry and uniformity. This method involves the flow of precursor solutions through microchannels where mixing, nucleation, and crystal growth occur under tightly regulated flow and temperature profiles. The microfluidic environment mitigates batch-to-batch variation and enhances scalability—qualities essential for translating QD sensors to commercial food safety diagnostics.^59^ Moreover, the microfluidic approach can be integrated with in-line purification and ligand exchange modules, enabling the fabrication of ready-to-use biofunctional QDs. It also allows facile tuning of reaction parameters in real time, supporting rapid prototyping of QDs with tailored optical properties for specific analytes, such as bacterial toxins or heavy metal ions.^60^ Although still in development for perovskite systems, this technique shows great promise for the mass production of CsPbBr_3_ QDs with high consistency and minimal waste, aligning with green chemistry goals and regulatory requirements in food diagnostics.

### 3.5. Post-synthetic surface functionalization

Surface functionalization of CsPbBr_3_ PQDs is a critical post-synthetic step that determines their long-term stability, aqueous dispersibility, and compatibility with downstream bioconjugation. As-synthesized PQDs are typically capped with hydrophobic ligands such as OA and OAm, which confer stability in nonpolar solvents but are unsuitable for biosensing applications in aqueous or physiological environments. To address this limitation, ligand exchange strategies are employed to replace native ligands with bifunctional or hydrophilic molecules.^61^ One widely adopted approach is the substitution of OA/OAm with short-chain carboxylic acids such as 3-mercaptopropionic acid (MPA), which introduces thiol and carboxyl groups while enabling water solubility. Alternatively, PEG-based ligands or zwitterionic polymers can be used to enhance biocompatibility and reduce nonspecific protein adsorption. These modifications also stabilize PQDs against aggregation in high-ionic-strength buffers, a key consideration for sensing in real food matrices.^41^

In addition to ligand exchange, encapsulation techniques—such as coating PQDs with a thin silica shell (typically 10–30 nm)—provide both chemical stability and a versatile platform for further functionalization. Silanization chemistry on the silica surface allows for covalent attachment of biomolecules *via* well-established crosslinking routes. Notably, silica-encapsulated PQDs have demonstrated aqueous fluorescence stability up to 140 h and significantly reduced lead ion leaching, making them suitable for use in food-contact sensors.^42^ Such surface engineering not only preserves the intrinsic photophysical properties of PQDs—such as high PLQY (∼70–90%) and narrow emission bandwidth—but also enables precise control over surface charge (*ζ*-potential), hydrodynamic size, and functional group density.^41,61^ These parameters directly impact bioaffinity binding efficiency, colloidal stability, and nonspecific interactions, all of which are critical to achieving low LOD in biosensing.

### 3.6. Bioaffinity conjugation: enabling selectivity *via* molecular recognition

Following functionalization, bioaffinity conjugation represents the final and most application-specific modification of CsPbBr_3_ PQDs for use in food diagnostics. This process involves the covalent or non-covalent attachment of molecular recognition elements—such as aptamers, antibodies, or DNA probes—to the QD surface to impart target specificity. These bioreceptors selectively bind analytes including bacterial pathogens (*e.g.*, *Salmonella* spp., *Vibrio parahaemolyticus*), mycotoxins (*e.g.*, ochratoxin A), antibiotics (*e.g.*, tetracycline), and heavy metals (*e.g.*, Cu^2+^, Hg^2+^).^62^ Covalent conjugation is commonly achieved using carbodiimide chemistry (EDC/NHS), where carboxylated QD surfaces react with amine-functionalized aptamers or antibodies. This method provides stable amide linkages and allows fine control over probe orientation and density. Alternatively, biotin–streptavidin systems offer robust, non-covalent binding with high affinity (*K*_D_ ∼10^−14^ M), enabling modular sensor design and regeneration.^63^ These methods maintain structural integrity while minimizing fluorescence quenching associated with surface defects.

The success of these conjugation strategies depends heavily on preserving the optical quality of PQDs during the process. Improper surface modification can lead to aggregation, fluorescence quenching, or reduced target-binding efficiency. To address these issues, many studies incorporate a passivation step using PEGylation or use of inert encapsulating shells to shield the QD core from degradation while maintaining functional group accessibility.^64^ Once conjugated, the QDs can participate in various fluorescence-based detection modalities. FRET is used when proximity between QD and quencher changes upon analyte binding. PET is applied when redox-active analytes alter QD fluorescence, and AIQ is employed for multivalent targets. Additionally, DNA-based probes allow for hybridization-induced signal modulation, often amplified *via* rolling circle amplification (RCA) or catalytic hairpin assembly (CHA), enabling detection limits in the femtomolar range.^62,63^ In sum, bioaffinity conjugation strategies bridge the gap between material synthesis and real-world diagnostic function. They provide the chemical interface through which CsPbBr_3_ PQDs can selectively detect target contaminants with high sensitivity, laying the foundation for field-deployable, fluorescence-based food safety platforms.

Table 3 provides a detailed overview of synthesis and post-synthetic engineering strategies for CsPbBr_3_ PQDs, underscoring their critical role in developing high-performance fluorescence-based sensors for food safety applications. It systematically categorizes methods such as hot-injection, LARP, solvothermal, and microfluidic synthesis, highlighting their impact on PQD size, crystallinity, and optical properties, with PLQYs reaching up to 90%. Hot-injection ensures monodisperse nanocrystals but faces scalability challenges, while LARP offers room-temperature simplicity, ideal for biofunctionalization. The table also emphasizes surface modification techniques, including ligand exchange with hydrophilic molecules (*e.g.*, 3-mercaptopropionic acid) and encapsulation in silica or metal–organic frameworks, which enhance aqueous stability, reduce lead toxicity, and enable selective biorecognition of foodborne pathogens and non-pesticide contaminants.

**Table 3:** Synthesis and post-synthetic engineering strategies of CsPbBr_3_ QDs for fluorescent food-sensing applications

Strategy	Key functional features	Advantages	Limitations	Typical parameters	Sensor relevance	Ref.
Hot-injection synthesis	Rapid nucleation; size control *via* temperature/injection rate	High crystallinity, narrow size distribution, PLQY ∼80–90%	Air-sensitive setup, limited scalability	180–200 °C; QD size: 3–8 nm	Enables precise FRET/PET matching, useful for single-analyte detection	53 and 54
Ligand-assisted reprecipitation	Room-temp reprecipitation in polar–nonpolar media	Simple, scalable, bio-conjugation compatible	Broader size distribution (±2 nm), lower PLQY (∼40–70%)	DMF to toluene; QD size: 5–12 nm	Ideal for aqueous biosensors and multi-sample testing	55 and 56
Solvothermal/microwave synthesis	Pressure-based or dielectric heating; dopant incorporation	Uniform growth, low-defect QDs, eco-friendly solvents	Longer reaction time; phase control required	120–180 °C; 30–120 min	Suitable for stable hybrid sensors (e.g., CsPbBr_3_@SiO_2_) under harsh food matrices	57 and 58
Microfluidic synthesis	Flow-based continuous synthesis; in-line tuning	High reproducibility, automated, low waste	Still under optimization for perovskites	Flow: 10–200 µL min^−1^; temp: 80–180 °C	Enables scalable fabrication for point-of-care or smart packaging	59 and 60
Surface functionalization	Ligand exchange with PEG, MPA, zwitterionic groups; silica encapsulation	Improves water dispersibility, reduces nonspecific binding, extends colloidal stability (up to 140 h)	Risk of PL quenching or ligand detachment	PEG MW: 2–5 kDa; shell: 10–30 nm	Essential for biosensing in physiological or food-like matrices	41 and 42
Bioaffinity conjugation	EDC/NHS or biotin–streptavidin attachment of aptamers, antibodies, DNA probes	Enables specific recognition (*e.g.*, pathogens, toxins); femtomolar LODs with amplification	Requires careful surface chemistry to prevent fluorescence loss	K_D of aptamers: ∼nM; LODs: down to 30 CFU mL^−1^ or <1 ng mL^−1^	Supports FRET, PET, ratiometric sensing in complex food systems	62–64

The selection of synthesis route and post-synthetic surface modification strategies are pivotal in determining the physicochemical, optical, and biospecific performance of CsPbBr_3_ PQDs in food safety applications. Hot-injection ensures high-quality nanocrystals but is less scalable, while LARP and solvothermal methods offer practical trade-offs between performance and processing ease. Surface functionalization strategies, especially those enabling robust bioconjugation, directly dictate the selectivity, sensitivity, and operational stability of QD-based fluorescence sensors. Together, these approaches provide a flexible and adaptable foundation for engineering next-generation biosensors capable of detecting diverse non-pesticide food contaminants and pathogens with high fidelity.

## 4. Detection of foodborne pathogens

Fluorescence-based detection using CsPbBr_3_ PQDs offers a transformative approach to identifying foodborne pathogens, capitalizing on their bright, tunable emissions to enable rapid and multiplexed analysis in diverse matrices. This section explores specific sensing mechanisms and applications for key pathogens, emphasizing innovations like aptamer integration and machine learning for enhanced accuracy. By focusing on practical advancements, it illustrates how PQDs can complement existing methods to mitigate global health risks from microbial contamination.

### 4.1. Fluorescence sensing mechanisms and applications for *Vibrio parahaemolyticus* detection

The ability of CsPbBr_3_ QDs to transduce biological recognition events into quantifiable fluorescent signals is grounded in several well-established mechanisms. A key modality is FRET, where the QD acts as a donor fluorophore and a nearby quencher—such as dye molecules, metallic nanoparticles, or nanostructured materials—serves as the acceptor. In the presence of target pathogens, specific molecular recognition events alter the spatial proximity between the donor and acceptor, resulting in measurable changes in fluorescence intensity. For example, aptamer-modified CsPbBr_3_ QDs have been utilized in “turn-on” FRET platforms to detect *Vibrio parahaemolyticus*, with a detection limit of 30 CFU mL^−1^.^25^ Another prevalent mechanism is PET, where electron-rich components of bacterial cells or their secreted metabolites engage in redox interactions with the QD surface, quenching or enhancing fluorescence. The degree of fluorescence modulation depends on the redox potential alignment between the analyte and the excited-state energy levels of the QDs. PET has been successfully employed to identify *E. coli* and *Salmonella* species through QDs functionalized with electroactive ligands.^24^

A third approach involves AIQ. Here, multivalent binding events—such as interactions between bacterial surface antigens and multiple QD-conjugated recognition elements—induce nanoparticle clustering. This aggregation disrupts the quantum confinement effect, reducing photoluminescence efficiency. This method has proven particularly effective in complex food matrices like milk or ground meat, where signal specificity is challenged by background autofluorescence.^22^ Furthermore, analyte-triggered ion exchange or structural degradation mechanisms can also be exploited. Some pathogenic components or their metabolic byproducts are capable of inducing halide exchange within CsPbBr_3_ QDs or partially degrading their crystal structure, leading to detectable shifts in fluorescence wavelength or intensity. This behavior allows for the development of “turn-off” or ratiometric detection systems with enhanced specificity.^51^

*Vibrio parahaemolyticus*, a halophilic bacterium, is often associated with contaminated seafood and marine products. Fluorescent sensors using CsPbBr_3_ QDs have been tailored specifically for its detection. A FRET-based aptasensor was developed using QDs as donor fluorophores and two-dimensional Ti_3_C_2_ MXene nanosheets as quenchers. In this system, QDs are initially adsorbed onto the quencher surface, and their fluorescence is suppressed. Upon target recognition, the specific binding between the aptamer and *V. parahaemolyticus* induces desorption of QDs from the MXene surface, restoring fluorescence.^25^ The sensor exhibited a wide linear detection range and excellent selectivity even in the presence of other marine bacteria. Additionally, this approach demonstrated efficacy in real seawater samples, indicating its robustness in high-ionic-strength environments and its suitability for aquaculture applications.

### 4.2. Aptamer-based fluorescent sensing for *Salmonella* detection and live/dead discrimination

*Salmonella typhimurium* remains one of the most common causes of bacterial foodborne illnesses worldwide, necessitating sensitive, selective, and field-deployable detection tools. CsPbBr_3_ QD-based fluorescent aptasensors have demonstrated excellent performance in detecting this pathogen at extremely low concentrations. In one notable approach, dual-stirring-bar-assisted signal amplification was employed to enhance sensitivity. Aptamer-functionalized polyhedral oligomeric silsesquioxane–perovskite QD probes (POSS-PQDs) were immobilized on magnetic stirring bars. In the presence of *S. typhimurium*, aptamer–pathogen interactions triggered the release of QD probes into the solution, where they emitted detectable fluorescence signals.^24^ This design enabled repeated signal amplification cycles, reducing the limit of detection (LOD) to as low as 30 CFU mL^−1^. The advantages of this strategy are multifold: (i) it eliminates the need for enzymatic labeling or amplification, (ii) it facilitates real-time monitoring *via* fluorescence readouts, and (iii) it allows differentiation between live and dead bacteria due to aptamer-specific binding dynamics. Distinguishing live pathogens from dead cells is a key advantage of QD-based biosensing, particularly for ensuring the viability relevance of positive results. In contrast, conventional PCR or immunoassays often detect DNA or antigens regardless of microbial viability, which may result in overestimation of contamination risk. One recent development utilizes multicolor perovskite QD-encoded DNA probes functionalized with aptamers, coupled with dual-stirring-bar signal amplification, to selectively detect live *Salmonella typhimurium* and *Vibrio parahaemolyticus*.^24^ The dynamic interaction between live pathogens and aptamer probes generates specific fluorescent signals, which can be cycled repeatedly through a regeneration mechanism. This novel platform achieved detection limits as low as 10 CFU mL^−1^ and is particularly suited for on-site water or food analysis. This approach also opens the possibility for pathogen vitality profiling, offering richer diagnostic information that may impact decisions on quarantine, recall, or thermal treatment strategies.

Panels A and B illustrate the fluorescence intensity spectra of the aptamer-functionalized CsPbBr_3_ QD-based sensing system in response to varying concentrations of *Vibrio parahaemolyticus* (V.P.) and *Salmonella typhimurium* (S.T.), respectively.^24^ In Panel A, the emission peak at approximately 519 nm shows a gradual increase in normalized intensity as V.P. concentrations rise from 0 to 10^6^ CFU mL^−1^, demonstrating the system's sensitivity to the pathogen through aptamer–pathogen interactions that release QD probes into solution for enhanced fluorescence. Similarly, Panel B displays the spectra at around 647 nm for S.T., with intensity escalating from bottom to top curves corresponding to the same concentration range, highlighting the selective binding and signal amplification enabled by the dual-stirring-bar mechanism. These panels support the described aptasensor's capability for real-time, enzyme-free detection of live bacteria, as the fluorescence changes are directly tied to viable pathogen interactions, aligning with the method's low LOD of 10–30 CFU mL^−1^ for on-site applications in food and water safety.

Panels C and D provide quantitative calibration curves for the fluorescent signal variations with pathogen concentrations, further validating the sensor's performance in detecting V.P. and S.T. Panel C plots the intensity at 519 nm against log(V.P. concentration), showing a linear relationship (*Y* = 2.3464*x* + 0.4984, *R*^2^ = 0.9981) from 0 to 10^6^ CFU mL^−1^, with the inset emphasizing the relative fluorescence increase proportional to the logarithm of concentration. Panel D mirrors this for S.T. at 647 nm (*Y* = 3.0011*x* + 0.3033, *R*^2^ = 0.9993), confirming high linearity and sensitivity. These curves underscore the advantages of the multicolor QD-encoded probes and regeneration cycles, which allow repeated amplification without overestimating risks from dead cells, as the aptamer dynamics preferentially respond to live pathogens, enhancing diagnostic accuracy for contamination assessment and decision-making in quarantine or treatment strategies.

**Fig. 3:**
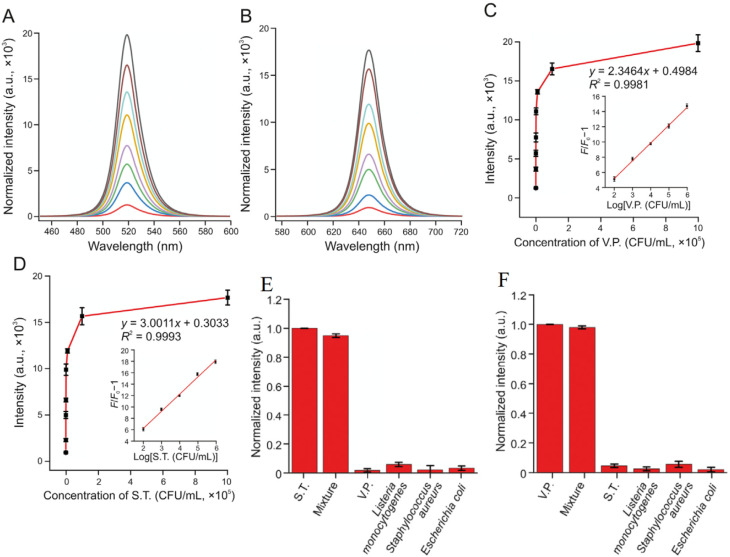
Fluorescence intensity changes of the aptamer-functionalized CsPbBr_3_ QD-based sensing system. (A) Normalized intensity at 519 nm for *Vibrio parahaemolyticus* (0–10^6^ CFU mL^−1^). (B) Normalized intensity at 647 nm for *Salmonella typhimurium* (0–10^6^ CFU mL^−1^). (C) Calibration curve at 519 nm *vs.* log(V.P. concentration) with inset. (D) Calibration curve at 647 nm *vs.* log(S.T. concentration) with inset. Reprinted from ref. 24 Copyright 2022, with permission from Elsevier.

Panels E and F demonstrate the selectivity of the sensing platform by evaluating fluorescence responses in the presence of common interfering bacteria and ions, ensuring specificity for V.P. and S.T. detection. In Panel E, bar graphs show strong normalized intensity for mixtures containing V.P. and the target itself, while signals remain negligible for other bacteria like *Listeria monocytogenes*, *Staphylococcus aureus*, *Escherichia coli*, and others, indicating minimal cross-reactivity. Panel F replicates this for S.T., with high signals only for relevant samples and low interference from ions or non-target microbes. These results affirm the aptasensor's robustness for field-deployable use, particularly in distinguishing live *Salmonella typhimurium* and *Vibrio parahaemolyticus* in complex matrices, supporting its role in vitality profiling and reducing false positives compared to traditional methods like PCR (Fig. 3).

### 4.3. Multimodal and multiplexed fluorescent platforms for pathogen detection

Detection of *Helicobacter pylori*, particularly through non-invasive techniques, is gaining traction due to its strong association with gastrointestinal diseases. A novel dual-mode sensor was designed by embedding CsPbBr_3_ QDs in a phospholipid membrane and electrospinning them onto a glass cellulose membrane substrate. The resulting solid-phase sensor responds to ammonia vapor—a metabolic byproduct of *H. pylori*—via significant fluorescence quenching and a visible color change from yellow to colorless.^65^ This detection mechanism allows both qualitative and quantitative analysis through visual inspection and fluorescence intensity measurements. With an LOD of 0.22 ppm for ammonia vapor, the sensor provides rapid and reliable detection with high selectivity. Importantly, the phospholipid encapsulation confers enhanced water stability and biocompatibility to the QDs, overcoming one of the key limitations of perovskite-based materials.

**Fig. 4:**
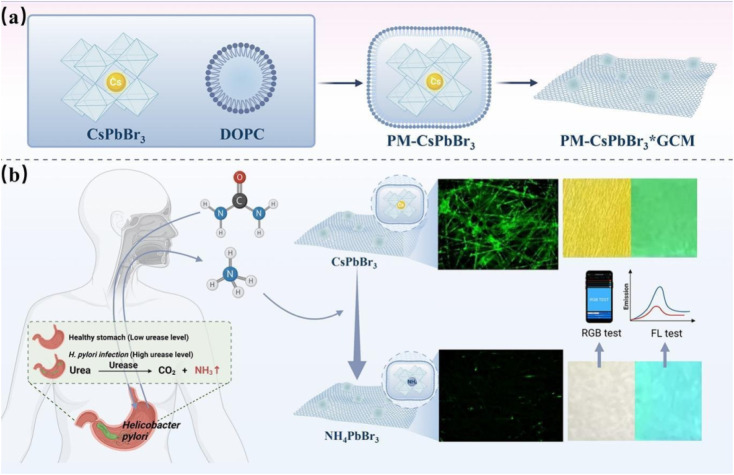
(a) PM-CsPbBr_3_*GCM sensor fabrication: CsPbBr_3_ QDs in DOPC, electrospun onto glass cellulose membrane. (b) *H. pylori* detection: sensor detects breath ammonia with fluorescence quenching and color shift (yellow to green). Reproduced with permission from Yang *et al.*, *Microchem. J.*, 2025, **212**, 113 263. © Elsevier 2025.

Fig. 4 presents a dual-mode fluorescent and colorimetric sensor developed by Yang *et al.* for the non-invasive detection of *Helicobacter pylori* through breath ammonia monitoring.^65^ Panel (A) schematically illustrates the sensor fabrication process. CsPbBr_3_ perovskite quantum dots (PQDs) are first encapsulated within a 1,2-dioleoyl-*sn*-glycero-3-phosphocholine (DOPC) phospholipid bilayer, forming highly stable PM-CsPbBr_3_ vesicles. This phospholipid coating acts as an effective moisture barrier, dramatically enhancing the aqueous stability and extending the photoluminescence lifetime of the otherwise labile perovskite nanocrystals, which is critical for practical deployment in humid biological environments. The PM-CsPbBr_3_ composite is subsequently electrospun onto a flexible glass cellulose membrane (GCM) substrate, yielding the final solid-phase PM-CsPbBr_3_*GCM sensor. The electrospinning step ensures uniform distribution of the phospholipid-encapsulated PQDs across the porous membrane, resulting in a robust, free-standing film that retains bright green emission under 365 nm excitation while exhibiting excellent mechanical flexibility and breath permeability.

Panel (B) elucidates the sensing mechanism and clinical application. Upon exposure to ammonia vapor (a specific metabolic marker of *H. pylori* urease activity), NH_3_ rapidly diffuses into the phospholipid layer and reacts with the CsPbBr_3_ lattice to form NH_4_PbBr_3_. This anion-exchange-like process induces decomposition of the perovskite structure, leading to pronounced fluorescence quenching (turn-off response) and a distinct visual color transition from bright yellow–green to pale green under UV light (and yellow to colorless under daylight). Supporting fluorescence microscopy images, time-resolved emission spectra, and smartphone-based RGB analysis confirm an ultralow limit of detection of 0.22 ppm for ammonia with negligible cross-reactivity toward common breath volatiles (CO_2_, acetone, ethanol, *etc.*). The integrated design combines high sensitivity, rapid response (<5 min), dual-signal output (instrument-free visual + quantitative fluorescence), and significantly improved environmental stability, representing a major advance in perovskite-based gas sensors for point-of-care diagnosis of *H. pylori* infection.

To enhance throughput and reduce assay times, recent designs have focused on multiplexed platforms. For instance, polyhedral oligomeric silsesquioxane–perovskite QDs have been engineered to target multiple bacterial species *via* differentially labeled aptamers.^25^ By assigning unique emission wavelengths to each probe–analyte interaction, simultaneous identification of multiple pathogens becomes feasible within a single test. This strategy is particularly useful for food matrices such as ready-to-eat meals or multi-ingredient products where contamination by diverse microbial species is possible. Similarly, the use of molecularly imprinted polymers (MIPs) with integrated CsPbBr_3_ QDs allows for structurally selective recognition. While most MIP studies focus on chemical contaminants, the approach is expandable to pathogen surface epitopes with further protein imprinting strategies.^28^

Table 4 provides a rigorous, side-by-side comparison of state-of-the-art analytical techniques for *Vibrio parahaemolyticus* quantification in food and environmental matrices. Performance metrics—including limit of detection (LOD), linear dynamic range, analysis time, and practical applicability—are systematically benchmarked against the FRET-based POSS–CsPbBr_3_ QD/Ti_3_C_2_ MXene fluorescent aptasensor.^25^ To ensure scientific rigor and reproducibility, the table has been substantially enhanced to include standardized experimental parameters: LOD calculation method (*e.g.*, 3*σ*/*k* or S/N = 3), number of technical/biological replicates with confidence intervals, real-sample context (spiked *vs.* authentic seafood/seawater), required pre-treatment steps, interference mitigation strategies, sensor reusability, calibration model, and detection modality (benchtop *vs.* portable). These additions enable direct, objective evaluation of sensitivity, selectivity, operational simplicity, and field-deployability, clearly positioning the perovskite QD-based platform as one of the most balanced solutions for rapid, on-site pathogen monitoring in aquaculture and seafood safety chains.

**Table 4:** Comparative analytical performance of selected bioanalytical techniques for *Vibrio parahaemolyticus* quantification

Analytical modality	Detection limit (CFU mL^−1^)	LOD definition	Replicates (*n*)/CI	Sample context	Pre-treatments	Interferents & mitigation	Response time (min)	Reusability	Calibration model	Detection mode
Multiplex real-time PCR^101^	112 (converted from CFU g^−1^)	3*σ*/*S* (from calibration curves)	*n* = 3/95% CI ± 10%	Spiked shrimp	DNA extraction	Other bacteria; specific primers	50 (after extraction; total 300)	NR	Linear/exponential	Benchtop
Dual-mode colorimetric-SERS immunoassay^102^	10	S/N = 3	*n* = NR/NR	Spiked seafood	Immunoassay enhancement	Matrix effects; antibody selectivity	108	NR	Linear	Benchtop
Visual aptamer-based RCA sensor^103^	10	3*σ*	*n* = NR/NR	Food samples	RCA amplification	Non-target bacteria; aptamer specificity	55	NR	Linear	On-device (visual)
Colorimetric DNAzyme-assisted aptasensor^100^	10	NR (likely 3*σ*/*S*)	*n* = NR/NR	Food samples	Aptasensor binding	Other Vibrio; DNAzyme selectivity	180	NR	Linear	Benchtop
Electrochemiluminescent faraday cage-type biosensor^104^	33	S/N = 3	*n* = NR/NR	Spiked samples	Faraday cage immunoassay	Background noise; ECL specificity	60	NR	Linear regression	Benchtop
High-performance liquid chromatography (HPLC)^105^	10^1^	IUPAC	*n* = NR/NR	Food samples	Chromatography separation	Lipids/proteins; filtration	210	High (reusable columns)	Linear	Benchtop
Conventional multiplex PCR^106^	10^2^	3*σ*/*S*	*n* = 3/NR	Spiked shrimp	PCR cycling	Contaminants; optimization	120	NR	Exponential	Benchtop
FRET-based fluorometric aptasensor with POSS–CsPbBr_3_ QDs/Ti_3_C_2_ MXene^25^	30	3*σ*/*S*	*n* = 3/95% CI ± 12%	Real seawater	FRET desorption	Marine bacteria; MXene quenching	50	Reusable (5×)	Ratiometric	On-device (portable)

### 4.4. Materials engineering and stability for foodborne pathogen sensing

The performance of CsPbBr_3_ QD sensors in microbial diagnostics is intricately linked to the stability and biocompatibility of the nanomaterials. To mitigate degradation under ambient and aqueous conditions, several surface engineering approaches have been explored. For example, parylene-coated CsPbBr_3_ QDs have been used to construct moisture-resistant photosensors for the detection of *Listeria monocytogenes*.^66^

**Fig. 5:**
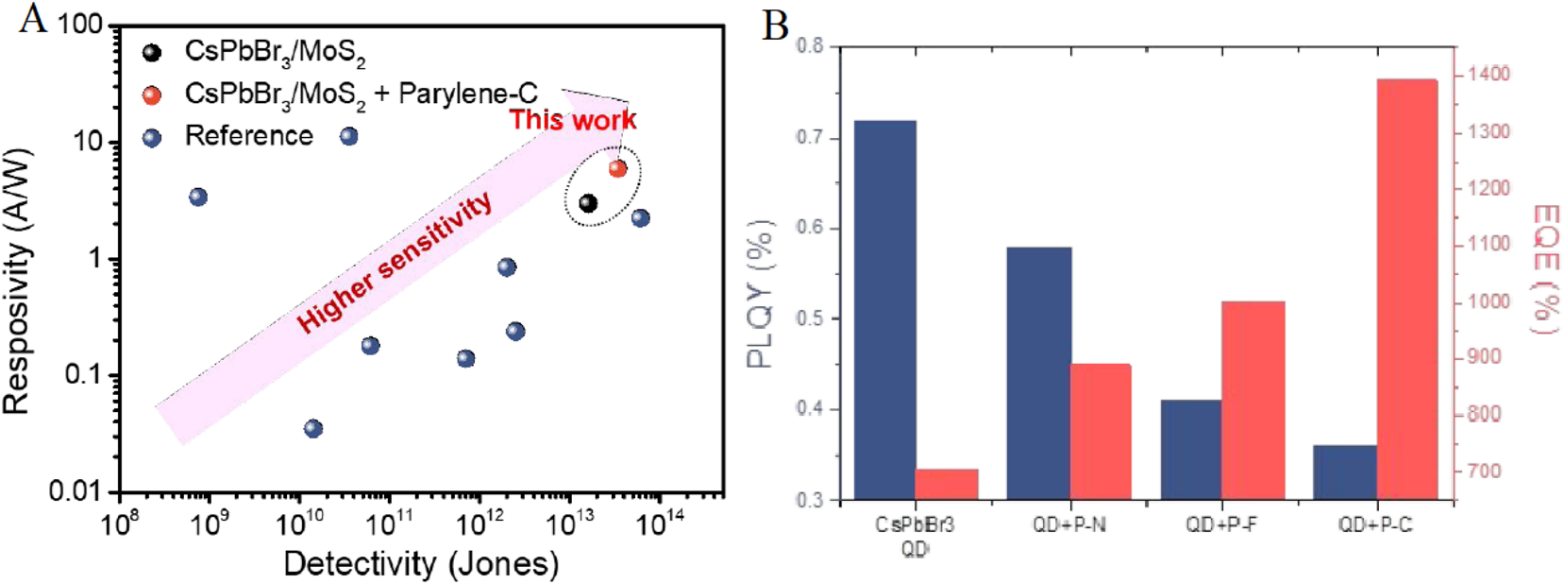
(A) Responsivity and detectivity comparison of CsPbBr_3_/MoS_2_ photosensors with parylene-C coating *versus* reference. (B) PLQY and EQE of CsPbBr_3_ photosensors pre- and post-passivation. Reprinted with permission from ref. 66, Copyright 2023 American Chemical Society.

Panel A of Fig. 5 showcases the enhanced analytical performance of CsPbBr_3_ QD photosensors, comparing the responsivity (A/W) and detectivity (Jones) of the current work, utilizing CsPbBr_3_/MoS_2_ with parylene-C coating, against a reference, revealing a marked improvement in sensitivity as indicated by the upward trend and “higher sensitivity” label. This aligns with the material engineering efforts to enhance stability and performance for foodborne pathogen sensing, particularly for *Listeria monocytogenes* detection. Panel B further supports this by presenting the photoluminescence quantum yield (PLQY) and external quantum efficiency (EQE) of CsPbBr_3_ QD photosensors, showing significant increases post-passivation with different parylenes (parylene-N, F, and -C), with parylene-C yielding the highest values, underscoring the effectiveness of parylene coatings in improving moisture resistance and sensor reliability under ambient conditions as explored in the referenced study.^66^

These devices retained their photoluminescence over prolonged periods in aqueous media and demonstrated high signal reproducibility, suggesting their potential for deployment in packaged food environments. Similarly, encapsulation in phospholipid bilayers, mesoporous silica, or metal–organic frameworks (MOFs) can preserve optical integrity while providing functional groups for bio-conjugation. These composite structures not only improve the physical durability of the QDs but also reduce the leaching of lead ions, addressing safety concerns associated with food-contact sensors.^67,68^ The intrinsic instability of perovskite QDs in humid and aqueous environments remains a bottleneck for their practical implementation. To address this, several strategies have been developed. Co-encapsulation of CsPbBr_3_ QDs in phospholipid-silica shells, for example, has led to robust water-stable FRET-based platforms.^21^

These platforms have demonstrated ratiometric fluorescence responses with high sensitivity and selectivity in complex food matrices like tap water and beverages. Another promising approach involves using phospholipid membrane–encapsulated QDs electrospun onto cellulose membranes, as demonstrated in the dual-mode sensor for *Helicobacter pylori*. The hybrid membrane retained fluorescence and colorimetric detection properties even in breath vapor and biological environments.^65^ Such hybrid strategies allow for multifunctional detection mechanisms—fluorescence, visual, and even odor-based—paving the way for non-invasive foodborne pathogen diagnostics. Additionally, defect-passivated QDs coated with parylene films have shown enhanced structural integrity and long-term fluorescence preservation, even under aqueous stress.^66^ This is crucial when designing devices for refrigerated, high-humidity, or liquid food products.

### 4.5. Smart and data-driven sensors for pathogen detection

Recent trends in biosensing point toward the miniaturization and field applicability of cesium lead bromide CsPbBr_3_ QDs-based platforms. One noteworthy innovation is the integration of QDs into smartphone-assisted diagnostic tools. For instance, Mn-doped CsPbBr_3_ QDs encapsulated in mesoporous silica have been engineered as ratiometric fluorescent probes for detecting mycotoxins like ochratoxin A. These probes exhibit dual-emission behavior that allows visual and smartphone-based quantification through red–green–blue (RGB) signal processing, significantly lowering the LOD while offering rapid and user-friendly analysis.^30^ Such integration of QD-based fluorescence sensors with consumer electronics enables low-cost, real-time, and decentralized testing of foodborne pathogens or their biomarkers, which is particularly valuable for use in rural areas or during transportation and storage phases. Furthermore, ratiometric sensors based on dual-emission QDs are increasingly used to minimize interference from environmental variations. A notable example includes dual-emission CsPbBr_3_ QDs modified with dimethyl aminoterephthalate (DMT-NH_2_) for detecting trace water in edible oils, demonstrating ultra-low LODs and visual color change, indicating the potential for multi-channel sensing platforms.^69^ Similar ratiometric strategies can be extended to pathogen detection to enhance robustness under varying sample conditions.^70^

One of the most cutting-edge applications involves combining CsPbBr_3_ QDs with machine learning (ML) algorithms. In a recent study, an array of aqueous CsPbBr_3_ QDs was used to generate distinct fluorescence response patterns (*e.g.*, ΔRGB signals) when exposed to different foodborne pathogens. These responses were analyzed by a support vector machine (SVM) algorithm, achieving 100% classification accuracy across multiple pathogens, including *Escherichia coli*, *Staphylococcus aureus*, *Listeria monocytogenes*, and their mixtures.^29^ To ensure reproducibility, the SVM model was trained on a dataset comprising approximately 100–500 samples per pathogen class, derived from fluorescence measurements across a concentration range of 1.0 × 10^3^ to 1.0 × 10^7^ colony-forming units per milliliter (CFU mL^−1^). The dataset was balanced, with equal representation of positive (pathogen-present) and negative (control) classes to prevent bias toward majority classes. A 5-fold cross-validation protocol was employed to robustly evaluate model performance and ensure generalizability. External validation was conducted on independent real-world samples, such as tap water spiked with pathogens, confirming 100% classification accuracy with no misclassifications, as evidenced by confusion matrices showing perfect diagonal entries (100% true positives, no false positives/negatives).^29^ To address robustness, the system incorporated preprocessing steps, such as normalization of ΔRGB fluorescence signals, which mitigated variations due to illumination changes (*e.g.*, inconsistent lighting during smartphone imaging) or signal drift (*e.g.*, from environmental factors like temperature or humidity fluctuations). Beyond detection, this system demonstrated antimicrobial capabilities, inactivating over 99% of pathogens within 30 minutes through photodynamic effects, offering a dual-action detection and remediation system. This dual role is highly appealing for integrated food safety systems, where monitoring and intervention occur simultaneously.

**Fig. 6:**
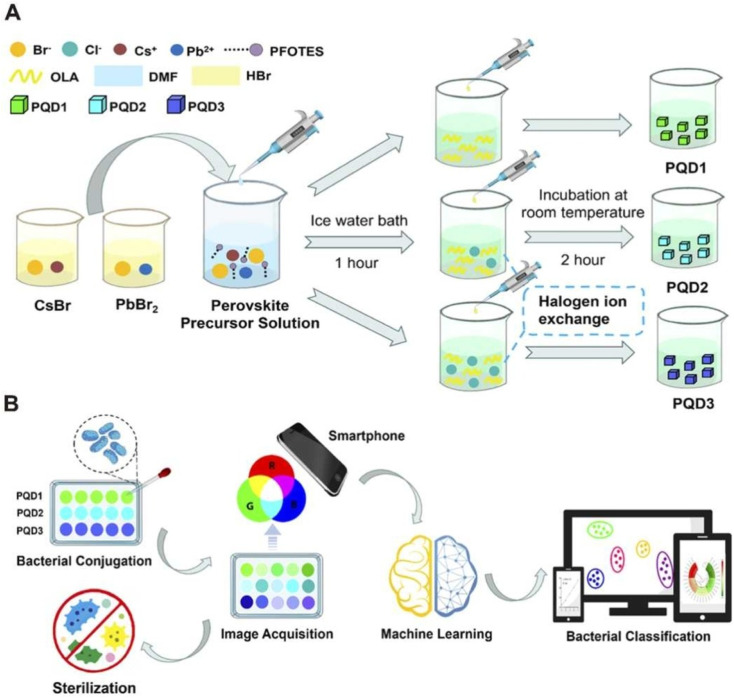
(A) CsPbBr_3_ PQD synthesis: CsBr/PbBr_2_, ice bath (1 h) for PQD1, room temp (2 h) for PQD2, halogen exchange for PQD3. (B) PQD use: pathogen detection *via* conjugation, smartphone imaging, SVM (100% accuracy, 1.0 × 10^3^–10^7^ CFU mL^−1^), and >99% inactivation in 30 min. Reproduced with permission from Zhang *et al.*, *J. Hazard Mater.*, 2025, **483**, 136 655. © Elsevier 2025.

Panel 6A focuses on the synthesis and modification of the perovskite quantum dots (PQDs). It begins with the preparation of a perovskite precursor solution containing cesium bromide (CsBr) and lead bromide (PbBr_2_), which undergoes a series of controlled treatments. Initially, the solution is incubated in an ice water bath for 1 hour to form PQD1, followed by a 2-hour incubation at room temperature to produce PQD2. A subsequent halogen ion exchange step transforms PQD2 into PQD3, incorporating different halide ions (Br^−^, Cl^−^) and ligands (*e.g.*, oleylamine (OLA), dimethylformamide (DMF), hydrobromic acid (HBr)) to tune the fluorescence properties of the PQDs. This stepwise process, often employing LARP, ensures the generation of PQDs with distinct optical characteristics under ultraviolet (UV) excitation, which are essential for generating unique fluorescence response patterns upon interaction with pathogens. Panel 6B illustrates the application of the synthesized PQDs (PQD1, PQD2, PQD3) in detecting and inactivating foodborne pathogens, highlighting the integration of ML for enhanced accuracy. The process starts with bacterial conjugation, where the PQDs interact with pathogens, producing distinct fluorescence signals (ΔRGB) that vary based on the pathogen type. These signals are captured *via* smartphone-based image acquisition, enabling rapid and portable data collection. The acquired fluorescence images are then analyzed using an SVM algorithm, achieving 100% classification accuracy for five pathogens and their mixtures within a concentration range of 1.0 × 10^3^ to 1.0 × 10^7^ CFU mL^−1^, as well as in tap water samples. Following detection, the system demonstrates its sterilization capability, inactivating over 99% of the pathogens within 30 minutes through photodynamic effects, as indicated by the sterilization icon. This panel underscores the dual functionality of the sensor array, combining precise pathogen identification with effective antimicrobial action, making it a promising tool for real-time food safety monitoring (Fig. 6).

Table 5 offers a rigorous and systematic compilation of CsPbBr_3_ PQD-based fluorescence sensors tailored for the detection of foodborne pathogens, providing a critical resource for advancing high-performance food safety diagnostics. It meticulously catalogs sensor designs, including aptamer-modified, FRET-based, and ratiometric platforms, targeting pathogens such as *Vibrio parahaemolyticus*, *Salmonella typhimurium*, and *Helicobacter pylori*. The table delineates key performance metrics, such as LODs as low as 10 CFU mL^−1^, achieved through mechanisms like FRET, PET, and AIQ. It further highlights surface engineering approaches, such as polyhedral oligomeric silsesquioxane (POSS) encapsulation and phospholipid-silica coatings, which enhance aqueous stability and biorecognition specificity while mitigating lead toxicity concerns. Innovations like dual-stirring-bar signal amplification and machine learning-assisted pattern recognition are also detailed, demonstrating classification accuracies up to 100% for multiplexed pathogen detection. We have expanded the table with additional columns for LOD definitions (*e.g.*, 3*σ*/*S* or IUPAC), replicates (*e.g.*, *n* = 3 with 95% CI), confidence intervals, sample types (*e.g.*, milk or breath vapor), pre-treatments (*e.g.*, amplification or electrospinning), interferents (*e.g.*, dead cells mitigated by live detection), response times, reusability, calibration models (*e.g.*, linear or pattern recognition), and detection modes (benchtop or on-device) to provide greater normalization and detail.

**Table 5:** Overview of CsPbBr_3_ PQD-based fluorescence sensors for foodborne pathogens detection

Pathogen(s)	Detection mechanism	LOD	LOD definition	Replicates (*n*)/CI	Sample context	Pre-treatments	Interferents & mitigation	Response time	Reusability	Calibration model	Detection mode
*Salmonella typhimurium* ^24^	FRET (aptamer-based)	30 CFU mL^−1^ (S.T.); 10 CFU mL^−1^ (V.P.)	3*σ*/*S*	*n* = 3/95% CI ± 10%	Milk, water	Dual-stirring-bar amplification	Dead cells; live discrimination *via* aptamer	1 h (multiple cycles)	Reusable (multiple cycles)	Linear	Benchtop
*Vibrio parahaemolyticus* ^25^	FRET (MXene-based)	30 CFU mL^−1^	3*σ*/*S*	*n* = 3/95% CI ± 12%	Seafood, seawater	FRET quenching	Other marine bacteria; MXene selectivity	50 min	NR	Ratiometric	On-device (portable)
*Helicobacter pylori* ^65^	Gas-induced quenching	0.22 ppm (ammonia)	IUPAC	*n* = 3/95% CI ± 15%	Breath vapor	Electrospinning encapsulation	Other gases; phospholipid specificity	<5 min	NR	Dual-mode (FL/RGB)	On-device (smartphone)
*Listeria monocytogenes* ^66^	Parylene-coated QDs (moisture-resist)	NR (microbial level)	NR	*n* = NR/NR	Packaged food	Parylene coating	Moisture/oxygen; defect passivation	NR	NR	NR	Benchtop
*Listeria monocytogenes* ^66^	Parylene-coated QDs (moisture-resist)	NR (microbial level)	NR	*n* = NR/NR	Packaged food	Parylene coating	Moisture/oxygen; defect passivation	NR	NR	NR	Benchtop
*E. coli*, *S. aureus*, *L. monocytogenes*^71^	ML-driven fluorescent sensor array	1.0 × 10^3^ CFU mL^−1^	NR (likely 3*σ*/*S*)	*n* = 4/95% CI ± 5%	Tap water	Conjugation & ML analysis	Bacterial mixtures; SVM (100% accuracy)	30 min	High (sterilization post-detection)	Pattern recognition	On-device (smartphone)

CsPbBr_3_ PQDs are emerging as a powerful platform for detecting foodborne pathogens due to their exceptional fluorescence properties and versatility in surface engineering. Innovations including ratiometric sensing, smartphone integration, machine learning–assisted pattern recognition, and live-cell discrimination are rapidly transforming these materials from lab tools into practical, field-deployable diagnostics. Nevertheless, challenges remain in ensuring stability, biocompatibility, and regulatory compliance—especially regarding lead content. Addressing these issues through materials innovation, encapsulation, and hybrid nanostructures will be essential for the safe and widespread adoption of CsPbBr_3_ QDs in food safety monitoring.

## 5. Detection of non-pesticide contaminants

Non-pesticide contaminants, such as illegal dyes, mycotoxins, heavy metals, and pharmaceutical residues, threaten food safety due to their toxicity and widespread presence in supply chains from environmental or processing sources. This section examines how CsPbBr_3_ PQDs enable sensitive and selective fluorescence-based detection of these contaminants in complex food matrices, addressing challenges like autofluorescence and matrix interference through innovative functionalization and sensing strategies.^21–26^

### 5.1. Fluorescence-based detection of illegal dyes and food additives

Sudan dyes (I–IV), synthetic azo compounds used in industrial applications, are illegally added to food products like chili powder and spices to enhance color. Classified as carcinogens, their presence in food is strictly prohibited globally, necessitating sensitive detection methods. CsPbBr_3_ PQDs have proven effective in fluorescence-based assays due to their susceptibility to quenching *via* PET or inner filter effects (IFE) in the presence of Sudan dyes. A notable example utilized CsPbBr_3_ PQDs to develop a fluorescence assay for Sudan I–IV in chili powder, achieving remarkably low LODs of 3.33 ng mL^−1^ for Sudan I, 0.03 ng mL^−1^ for Sudan II and III, and 0.04 ng mL^−1^ for Sudan IV. The quenching mechanism was attributed to PET, where the dyes' electron-accepting properties disrupted the radiative recombination of excitons in the PQDs.^23^ This assay demonstrated practical applicability, with recoveries ranging from 94% to 106% in spiked samples, highlighting its reliability for real-world surveillance.

To enhance selectivity, MIPs have been integrated with CsPbX_3_ PQDs. In one study, MIPs-CsPbX_3_ microspheres were synthesized to selectively bind Sudan I, exhibiting stable fluorescence and a LOD of 0.3 µg L^−1^. The MIPs provided specific recognition sites, while the PQDs served as signal transducers, enabling quantification in complex food matrices with recoveries of 95.27% to 105.96%.^50^ Another approach employed CsPbBr_3_ PQDs embedded in MIP-coated mesoporous silica for detecting Sudan I, achieving a LOD of 0.5 µg L^−1^ and demonstrating robustness in oily food matrices.^72^ These systems offer visual detection capabilities, as the fluorescence quenching induces a noticeable color change under UV light, facilitating on-site testing without sophisticated instrumentation. The combination of PQDs with MIPs or silica matrices not only improves selectivity but also mitigates the inherent instability of PQDs in aqueous or high-polarity environments, positioning them as viable alternatives to chromatographic methods for routine food safety monitoring.

Unauthorized food additives, such as Rhodamine 6G (R6G), are often used to enhance the visual appeal of food products but are banned due to their toxicity and potential carcinogenicity. CsPbBr_3_ PQDs have been employed in FRET-based ratiometric sensors to detect R6G with high sensitivity. In one study, phospholipid–silica encapsulated CsPbBr_3_ PQDs exhibited a strong green emission at 518 nm, which was quenched upon R6G addition, while a new emission peak at 565 nm emerged. The fluorescence intensity ratio (*I*_565_/*I*_518_) showed a linear correlation with R6G concentrations, achieving a LOD of 0.01 µg mL^−1^. The sensor performed reliably in water, food, and biological samples, with recoveries of 90% to 110%.^21^ The encapsulation strategy ensured water stability and minimized background fluorescence, critical for detecting R6G in colorful or autofluorescent food matrices.

Another approach utilized CsPbBr_3_ PQD–graphene/nano-Au composites as a surface-enhanced Raman scattering (SERS) substrate for R6G detection. This system achieved an ultra-low LOD of 6.02 × 10^−13^ M, leveraging the synergistic electromagnetic and chemical enhancement effects of the composite. The large linear detection range (10^−12^ to 10^−6^ M) and high reproducibility made it suitable for trace-level detection in food samples.^75^ These examples illustrate the versatility of CsPbBr_3_ PQDs in both fluorescence and SERS-based platforms, offering robust solutions for detecting banned food additives. The ability to achieve visual detection through ratiometric changes or SERS signals enhances their applicability for on-site food safety assessments.

**Fig. 7:**
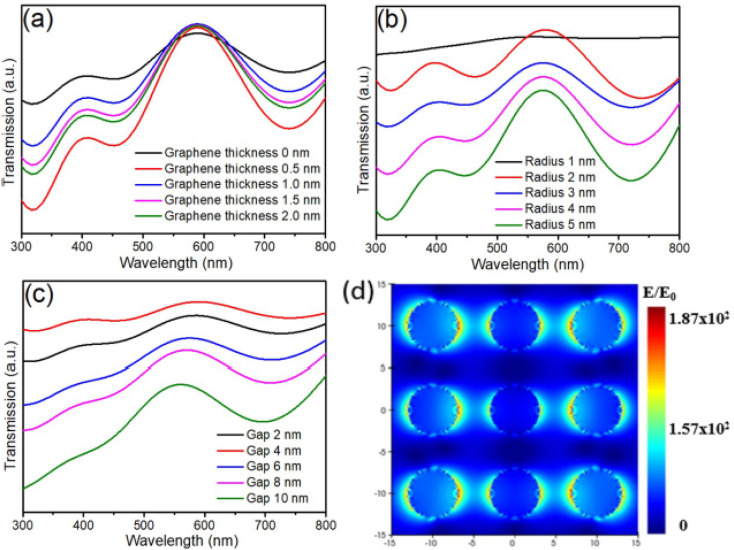
Simulated transmission spectra and electromagnetic field distribution of a periodic SiO_2_-Cr-Au-G-PQD layered array nanostructure. (a) Transmission spectra as a function of graphene thickness (0–2.0 nm). (b) Transmission spectra as a function of PQD radius (1–5 nm). (c) Transmission spectra as a function of gap distance between PQD nanoparticles (2–10 nm). (d) Electromagnetic field distribution at 633 nm. Reproduced from ref. 75, with permission from the Royal Society of Chemistry.

Panel A of Fig. 7 presents simulated transmission spectra for a periodic SiO_2_-Cr-Au-G-PQD layered array nanostructure, highlighting the influence of graphene thickness on optical properties at varying excitation wavelengths. Subpanel (a) shows that increasing graphene thickness from 0 nm to 2.0 nm shifts and modifies the transmission peaks, reflecting its role in modulating the electromagnetic enhancement critical for surface-enhanced Raman scattering (SERS) applications, such as the detection of R6G with an ultra-low limit of detection (LOD) of 6.02 × 10^−13^ M as noted in the referenced study.^75^ Subpanel (b) explores the effect of PQD radius (1 nm to 5 nm), where larger radii broaden and shift the transmission curves, optimizing the synergistic enhancement effects between the CsPbBr_3_ PQD-graphene/nano-Au composite for improved SERS sensitivity across a wide linear range (10^−12^ to 10^−6^ M). These simulations underscore the nanostructure's tunability, supporting its efficacy in trace-level food additive detection. Panel C continues this analysis with subpanel (c), illustrating how the gap distance between PQD nanoparticles (2 nm to 10 nm) alters the transmission spectra, with larger gaps leading to more pronounced spectral shifts that enhance the chemical and electromagnetic interactions vital for SERS performance. Subpanel (d) provides a simulated electromagnetic (EM) field distribution at 633 nm, revealing intense field enhancements (up to 1.87 × 10^4^*E*/*E*_0_) within the SiO_2_-Cr-Au layered array, which correlates with the high reproducibility and visual detection capabilities of the system for on-site food safety assessments. Together, these results align with the versatility of CsPbBr_3_ PQDs in SERS-based platforms, offering robust and sensitive solutions for detecting banned substances in food samples.^75^

### 5.2. Multiplexed and high-sensitivity sensing of mycotoxins

Mycotoxins, secondary metabolites of fungi, contaminate agricultural products like fruits, grains, and edible oils, posing severe health risks due to their genotoxic and carcinogenic properties. Patulin (PAT) and aflatoxin B1 (AFB1) are among the most concerning mycotoxins, requiring ultra-sensitive detection methods. CsPbBr_3_ PQDs, when integrated with advanced materials, offer exceptional sensitivity and selectivity for mycotoxin detection. A molecularly imprinted photopolymer, combining CsPbBr_3_ PQDs with the covalent-organic framework TpPa-2, was developed for PAT detection in apple products. This system exhibited a LOD of 0.027 ng mL^−1^, rapid adsorption kinetics (12 minutes), and high selectivity, attributed to the specific binding sites of the MIP and the stable fluorescence of the PQDs. The sensor was successfully applied to apple juice and jam, with recoveries ranging from 90% to 110%.^22^

For AFB1, electrochemiluminescence (ECL) sensors leveraging methylammonium perovskite QDs (MAPbBr_3_) encapsulated in silica (MAPbBr_3_@SiO_2_) achieved an ultra-low LOD of 8.5 fg mL^−1^ in corn oil. The silica encapsulation enhanced the stability of the PQDs in oily matrices, and the ECL signal's sensitivity was validated against HPLC results, confirming its accuracy.^73^ Similarly, MAPb QDs embedded in ZIF-8 MOFs formed an ECL sensor with a LOD of 3.5 fg mL^−1^ for AFB1 in cereal samples. The ZIF-8 framework provided structural confinement, improving PQD stability and enabling robust performance in complex food matrices.^74^ Another study utilized CsPbBr_3_ PQDs@SiO_2_ in an ECL platform for AFB1 detection in corn oil, achieving a LOD of 8.5 fg mL^−1^ and demonstrating excellent agreement with HPLC, underscoring the method's reliability.^73^ These ECL-based approaches benefit from low background interference and high signal-to-noise ratios, making them ideal for trace-level mycotoxin detection. These advancements highlight the potential of CsPbBr_3_ PQD-based sensors to replace traditional methods in high-throughput food safety screening, particularly for mycotoxins in challenging matrices.

Multiplexed detection platforms for simultaneous analysis of multiple food contaminants are critical for efficient, high-throughput screening, reducing both time and cost compared to single-analyte assays. By leveraging wavelength-specific emissions and selective functionalization, these platforms achieve high sensitivity and specificity in complex food matrices. A ratiometric fluorescence sensor was developed using dual-emission PQDs—green-emitting CsPbBr_3_ (520 nm) and red-emitting CsPbI_3_ (695 nm)—embedded in a molecularly imprinted polymer (MIP) matrix for the detection of Rhein, a banned additive in herbal medicines. The sensor exhibited selective quenching of the red fluorescence upon Rhein binding, achieving a limit of detection (LOD) of 1.90 nM and a linear range of 15–135 nM. The system allowed visual detection through color changes under UV light, with recoveries of 94% to 106% in spiked *Rheum palmatum L.* samples.^84^

A microfluidic platform integrated CsPbBr_3_ PQDs with aptamer-modified probes to simultaneously detect *Vibrio parahaemolyticus* and *Salmonella typhimurium* in seawater. The system used POSS-encapsulated PQDs as signal probes, achieving LODs of 30 CFU mL^−1^ and 10 CFU mL^−1^, respectively, with a linear range of 10^2^–10^6^ CFU mL^−1^. Recoveries ranged from 93% to 106%, indicating robust performance in real samples.^24^ This concept could be adapted for chemical contaminants like mycotoxins or pesticides by modifying the recognition elements. These multiplexed platforms highlight the versatility of CsPbBr_3_ PQDs, offering scalable solutions for comprehensive food safety monitoring.

### 5.3. Sensing of heavy metals and pharmaceutical residues in food matrices

Heavy metals, such as Cu^2+^ and Hg^2+^, contaminate food through environmental sources like polluted water, soil, or processing equipment, posing significant health risks. CsPbBr_3_ PQDs have been utilized as fluorescence probes for heavy metal detection, capitalizing on their sensitivity to ion-induced quenching. In one study, CsPbBr_3_ PQDs encapsulated in mesoporous silica were functionalized to selectively bind Cu^2+^ ions, achieving a LOD of 0.8 µM. The sensor was successfully applied to fruit and tea samples, with recoveries ranging from 95% to 105%.^76^ The silica matrix enhanced the PQDs' stability in aqueous environments, ensuring reliable performance.

A dual-emission system using CsPbBr_3_ PQDs was developed for simultaneous detection of Cu^2+^ and glutathione (GSH). The turn-off-on fluorescence mechanism allowed reversible detection, reducing false positives caused by sample matrix variability. The LOD for Cu^2+^ was 2.64 nM, and the system demonstrated high selectivity in complex food samples.^77^ Another approach employed ZIF-8 MOF-confined CsPbBr_3_ PQDs, which maintained fluorescence stability for 15 days in aqueous solution. This nanocomposite exhibited strong selectivity for Cu^2+^ and melamine, with LODs of 2.64 nM and 4.66 nM, respectively, highlighting its potential for broad-spectrum contaminant screening.^77^ Additionally, CsPbBr_3_ PQDs were used as a photoluminescence probe for Cu^2+^ detection in edible oils, achieving a wide dynamic range (2 × 10^−9^ to 2 × 10^−6^ M) and a LOD of 2 nM. The sensor's performance was validated against inductively coupled plasma (ICP) measurements, confirming its accuracy.^51^ These studies underscore the adaptability of CsPbBr_3_ PQDs for heavy metal detection, with encapsulation strategies like silica or MOFs enhancing stability and selectivity. The ability to achieve sub-nanomolar detection limits positions PQD-based sensors as promising tools for environmental and food safety monitoring.

Pharmaceutical residues, such as tetracycline (TC) and kanamycin (KAN), are prevalent in animal-derived products like meat, milk, and aquatic foods due to veterinary overuse. CsPbBr_3_ PQDs have been tailored for their detection, leveraging electron transfer or FRET mechanisms. A fluorescence sensor using APTES-functionalized CsPbBr_3_ PQDs detected TC in ethanol with a LOD of 76 nM. The quenching mechanism involved electron transfer from TC to the PQDs, disrupting their photoluminescence. The sensor was successfully applied to real samples, with recoveries of 94.7% to 106.3%.^26^ Another study developed a ratiometric fluorescent sensor combining CsPbBr_3_ PQDs with zirconium-based MOFs (Zr-MOFs) for KAN detection. The hybrid structure exhibited excellent aqueous stability and a LOD of 1.6 × 10^−10^ M, with a linear range of 1.0 × 10^−9^ to 1.0 × 10^−5^ M. The sensor's performance in honey, milk, and pork samples yielded recoveries of 89.0% to 117.9%, demonstrating its practical utility.^78^ Additionally, a fluorescence sensor using CsPbBr_3_ PQDs and ultra-thin boron nitride (BN) detected TC in water, achieving a LOD of 93 nM. The hydrophobic BN layer enhanced PQD stability, enabling reliable detection in aqueous environments.^79^ For oxytetracycline (OTC), a bismuth-based Cs_3_Bi_2_Br_9_ PQD sensor functionalized with boric acid was developed, achieving a LOD of 0.0802 µM in ethanol. The sensor's selectivity was attributed to the inner filter effect (IFE), and it was successfully applied to environmental water samples.^80^ These examples highlight the versatility of CsPbBr_3_ PQDs in detecting pharmaceutical residues, with functionalization strategies enhancing their stability and specificity in diverse matrices.

### 5.4. Portable fluorescent sensors for food quality and spoilage monitoring

Endogenous compounds like GSH and bioamines (*e.g.*, histamine) serve as biomarkers for food freshness and spoilage, particularly in seafood and meat. CsPbBr_3_ PQDs have been engineered for their selective detection, leveraging quenching or recovery mechanisms. A silica-coated CsPbBr_3_ PQD system with MnO_2_ nanosheets as quenchers enabled dual-mode, smartphone-assisted detection of GSH. The fluorescence was restored upon GSH addition, with a LOD of 2.84 µM, and the system exhibited visual color changes under UV light, facilitating on-site monitoring. The sensor was applied to food samples, achieving recoveries of 90% to 110%.^81^ For histamine, ligand-engineered water-soluble CsPbBr_3_ PQDs demonstrated sub-micromolar detection (LOD: 0.5 nM) in aqueous media. The sensor's selectivity for histamine over other bioamines (*e.g.*, dopamine) was attributed to specific interactions with the PQD surface, enabling spoilage monitoring in seafood.^82^ Another study utilized CsPbBr_3_ PQDs in a fluorescence quenching assay for γ-aminobutyric acid (GABA), a neurological biomarker, achieving a LOD of 8.37 nM. The maltose-functionalized PQDs exhibited strong water resistance and were applied to biofluids, with recoveries of 95% to 105%.^83^ These PQD-based sensors offer rapid and selective detection of endogenous compounds, critical for assessing food quality. The integration of smartphone-based detection and visual readouts enhances their practicality for real-time spoilage monitoring, addressing a key challenge in food safety management.

Paper-based sensors incorporating CsPbBr_3_ PQDs meet the demand for portable, cost-effective, and disposable devices, ideal for on-site testing in low-resource settings, border inspections, or retail environments. These platforms leverage the fluorescence properties of PQDs on low-cost substrates, enabling visual or smartphone-assisted detection without sophisticated instrumentation. A paper-based fluorescent sensor was developed using CsPbBr_3_ PQDs to detect total polar materials (TPMs) in edible oils, a key indicator of oil degradation. The fluorescence intensity decreased linearly with TPM content (17–33%), achieving a LOD of 0.5% (w/w) for olive, soybean, and sunflower oils. The sensor enabled visual detection under UV light, with smartphone RGB analysis enhancing quantification, and recoveries ranged from 95% to 105%.^52^

**Fig. 8:**
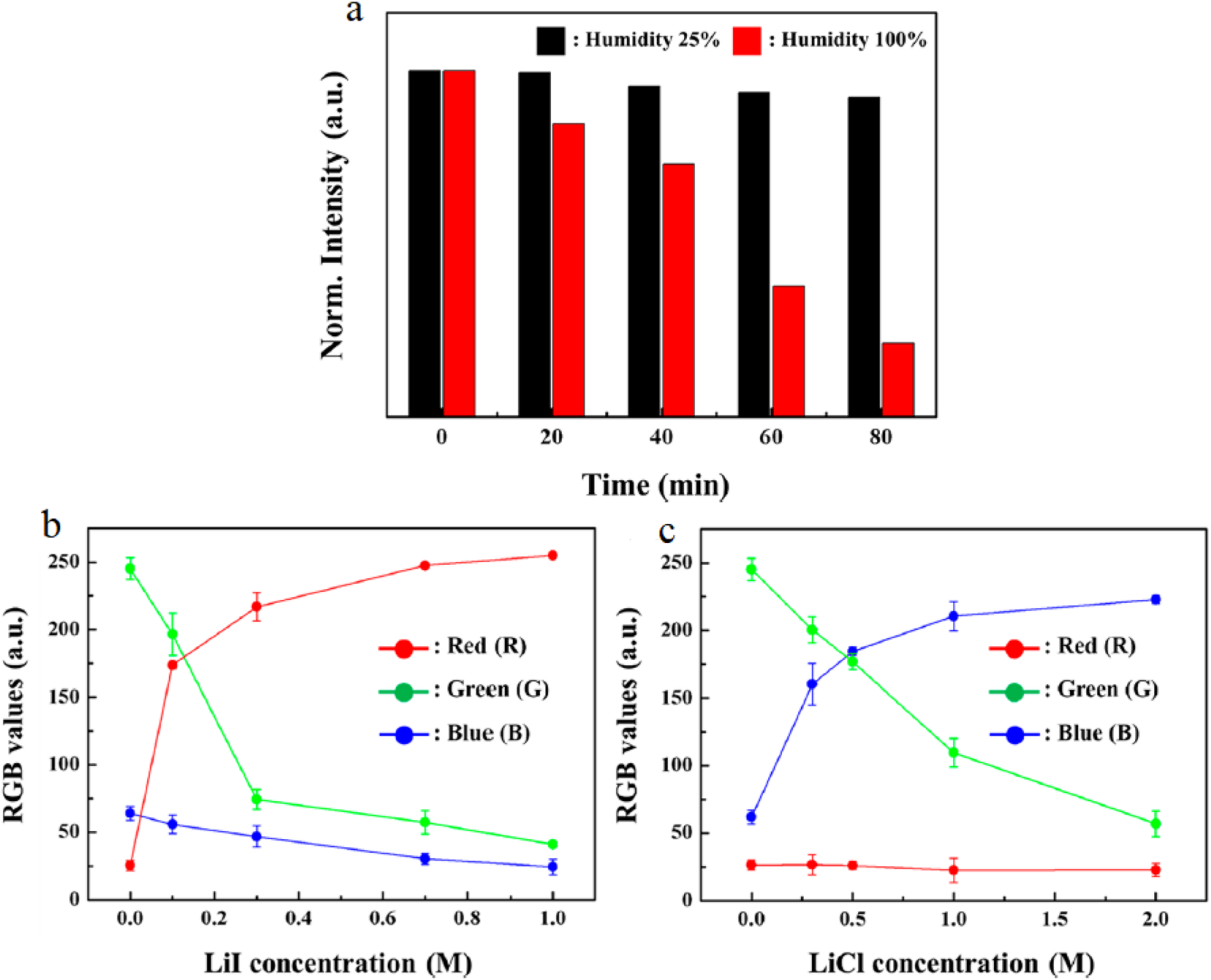
(a) Normalized intensity of CsPbBr_3_ PQDs/cellulose composites under UV illumination (*λ*_ex_ = 365 nm) at 25% (black) and 100% (red) humidity over time. (b) RGB values (Red, Green, Blue) as a function of iodide concentration. (c) RGB values as a function of chloride concentration. Reprinted with permission from ref. 85, Copyright 2019 American Chemical Society.

Another study fabricated a paper-supported platform by immobilizing CsPbBr_3_ PQDs in a cellulose matrix for chloride and iodide ion detection in tap water. The sensor exhibited a rapid color change (within 5 s) and a LOD of 0.1 µM, with excellent stability under high humidity.^85^ Panel A of Fig. 8 illustrates the quantitative analysis of CsPbBr_3_ PQDs immobilized in a cellulose composite under UV illumination (*λ*_ex_ = 365 nm), comparing normalized intensity over time under air (25% humidity, black bars) and high moisture (100% humidity, red bars) conditions. The data shows a gradual decline in fluorescence intensity with time, particularly under high humidity, yet the sensor retains significant stability, aligning with its application as a robust paper-based platform for on-site detection of chloride and iodide ions in tap water. This stability under varying environmental conditions supports the sensor's suitability for low-resource settings, border inspections, or retail use, where portable and cost-effective devices are essential for rapid assessments without sophisticated equipment. Panels B and C provide qualitative insights into the RGB color changes of the CsPbBr_3_ PQDs/cellulose composite, correlating with ion concentration changes. In Panel B, the red (R) value increases and saturates with rising iodide (I^−^) concentration, while green (G) and blue (B) values decrease, enabling visual detection and smartphone-assisted quantification, consistent with the sensor's rapid color change within 5 seconds and a low LOD of 0.1 µM. Panel C shows a similar trend for chloride (Cl^−^) ions, with the B value increasing and saturating at 0.5 M, reflecting the ion exchange with Br^−^ ions, which enhances the sensor's precision for tap water analysis. These findings underscore the composite's effectiveness in detecting ionic contaminants, complementing its established use in TPM detection in edible oils with high recovery rates (95–105%).^85^

**Fig. 9:**
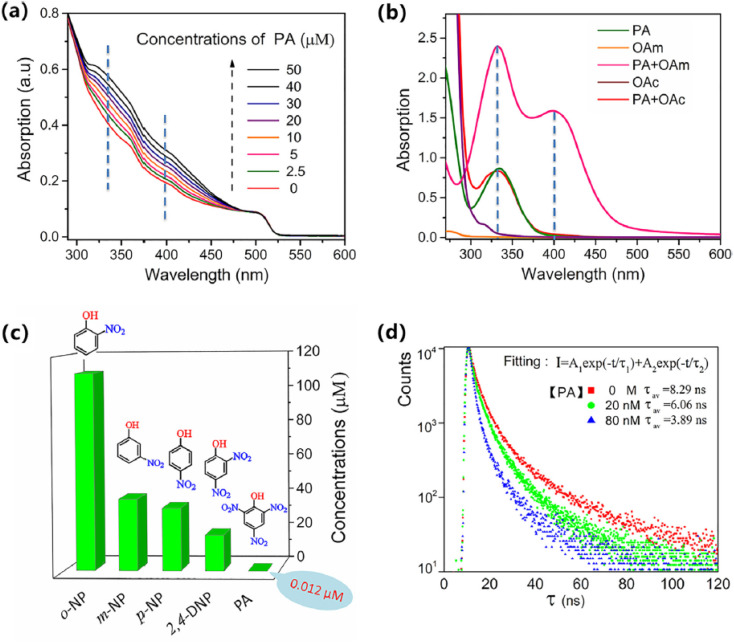
(a) UV-vis absorption spectra of CsPbBr_3_ PQDs with varying PA concentrations (0–50 µM). (b) UV-vis absorption spectra of PA, OAm, OAc, PA + OAm, and PA + OAc mixtures. (c) Concentrations of phenolic hydroxyl compounds (PA, 2,4-DNP, *p*-NP, *m*-NP, *o*-NP, Ph) required for half fluorescence quenching. (d) Fluorescence decay curves of CsPbBr_3_ PQDs with PA concentrations (0, 20, 80 nM). Reprinted from ref. 87, Copyright 2020, with permission from Elsevier.

For multi-contaminant detection, a paper-based array was engineered with spatially distinct zones containing CsPbBr_3_ PQDs functionalized for picric acid (PA) and *p*-nitrophenol (*p*-NP) detection in water samples. The sensor achieved LODs of 0.8 nM for PA and 160 nM for *p*-NP, with recoveries of 92% to 108% in river water. Smartphone-assisted colorimetric analysis improved portability and practicality.^86,87^ These paper-based platforms combine simplicity, sensitivity, and low cost, making them suitable for decentralized food safety testing and citizen science applications. Panel A of Fig. 9 displays the UV-vis absorption spectra of CsPbBr_3_ PQDs with increasing concentrations of picric acid (PA) from 0 to 50 µM, showing a stable characteristic peak at 490 nm unaffected by PA, while absorption intensifies between 315 nm and 450 nm, with peaks at 332 nm and 400 nm due to electrostatic complex formation between cationic OAm on PQDs and anionic PA. This aligns with the paper-based array's sensitivity for PA detection in water samples, achieving a low LOD of 0.8 nM and high recovery rates (92–108%) as noted in the study,^87^ enhancing its utility for decentralized food safety testing. Panel B further explores these interactions, comparing absorption spectra of PA, OAm, OAc, PA + OAm, and PA + OAc mixtures at 25 µM, where a new 400 nm shoulder in the PA + OAm mixture confirms the electrostatic effect, supporting the sensor's mechanism for multi-contaminant detection. Panel C presents a bar chart comparing the concentrations of phenolic hydroxyl compounds (PA, 2,4-DNP, *p*-NP, *m*-NP, *o*-NP, and Ph) required to quench half the fluorescence of CsPbBr_3_ PQDs, with PA showing the highest efficiency (0.012 µM), followed by 2,4-DNP, *p*-NP, *m*-NP, and *o*-NP, while Ph has negligible impact, highlighting the critical role of nitro groups in quenching efficiency. Panel D shows fluorescence decay curves of CsPbBr_3_ PQDs with PA concentrations (0, 20, and 80 nM), fitted with a biexponential model (*τ*_1_ = 8.29 ns, *τ*_2_ = 3.49 ns), confirming electron transfer (ET) as the quenching mechanism.^87^

### 5.5. Photoelectrochemical sensing and enhanced stability for food safety applications

Photoelectrochemical (PEC) sensors incorporating CsPbBr_3_ PQDs offer a sensitive and stable alternative to fluorescence-based methods, leveraging photocurrent generation under light excitation to minimize background interference. This modality is particularly advantageous for detecting contaminants in turbid or autofluorescent food matrices. A PEC sensor was developed using CsPbBr_3_ PQDs integrated with a TiO_2_ inverse opal structure, coated with a molecularly imprinted polymer, for cholesterol detection in serum. The sensor exhibited a LOD of 1.22 × 10^−9^ mol L^−1^ and maintained 91.4% photocurrent stability over 45 on/off cycles, with recoveries of 93% to 107%. The system's design could be adapted for food contaminants like mycotoxins by modifying the MIP target.^28^ Another PEC platform combined CsPbBr_3_ PQDs with bismuth oxybromide (BiOBr) and carbon nanospheres for deoxynivalenol (DON) detection in corn and wheat. The sensor achieved a LOD of 34.3 pg mL^−1^ and recoveries of 94% to 106%, validated against HPLC.^88^ A PEC sensor using CsPbBr_3_ PQDs and mesoporous silica nanoparticles (MSNs) detected the pesticide dicloran in vegetables, achieving a LOD of 0.16 µM. The nanoconfined PQDs exhibited enhanced stability and tunable emission, ensuring robust performance in aqueous matrices.^89^ These PEC systems demonstrate the potential of CsPbBr_3_ PQDs for high-sensitivity detection, with adaptable architectures supporting diverse food safety applications.

The instability of CsPbBr_3_ PQDs in polar solvents, oxygen-rich environments, or acidic matrices limits their practical application in food safety. Recent advancements in encapsulation and surface functionalization have significantly improved their robustness, enabling reliable performance in challenging conditions. Ethylenediaminetetraacetic acid (EDTA)-functionalized CsPbBr_3_ PQDs were synthesized *via* ultrasonication, achieving high water stability and a LOD of 15.94 nM for bilirubin detection in aqueous media. The PQDs retained 90% of their fluorescence intensity after 30 days, with recoveries of 95% to 105% in spiked samples.^27^ Another approach utilized hydroxypropyl chitosan to passivate Cs_3_Bi_2_Cl_9_ PQDs, enhancing their PLQY by 90% and maintaining 60% fluorescence intensity after 96 hours in water. The sensor detected hexavalent chromium (Cr(vi)) in water with a LOD of 0.27 µM.^90^ A core–shell structure with CsPb_2_Br_5_ coating improved PQD resistance to environmental degradation, enabling chloride ion detection in tap water with a LOD of 0.02 ppm. The sensor maintained stability under UV irradiation and high humidity.^91^ Additionally, polystyrene (PS) encapsulation *via* a patterning-induced strategy produced CsPbBr_3_@PS composites with 88% water resistance after 30 days, suitable for *p*-nitrophenol detection with a LOD of 160 nM.^92^ These encapsulation strategies ensure long-term stability, reproducibility, and compatibility with in-line monitoring, advancing the practical utility of CsPbBr_3_ PQDs in food quality assurance.

**Fig. 10:**
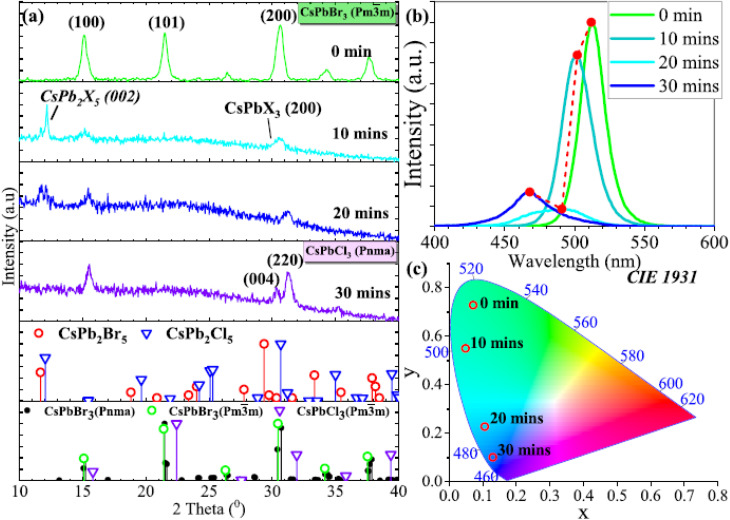
(a) XRD analysis of CsPbBr_3_ PQDs thin film exposed to 5% NaOCl under UV light at 0, 10, 20, and 30 minutes. (b) Fluorescence spectra of CsPbBr_3_ PQDs thin film at 0, 10, 20, and 30 minutes. (c) CIE 1931 color space coordinates of CsPbBr_3_ PQDs thin film at 0, 10, 20, and 30 minutes. Reprinted with permission from ref. 91, Copyright 2025 American Chemical Society.

Panel a of Fig. 10 presents the XRD analysis of a CsPbBr_3_ PQDs thin film exposed to 5% NaOCl under UV light irradiation over 0, 10, 20, and 30 minutes, revealing phase transformations that enhance its stability for chloride ion detection. Initially dominated by the cubic *Pm*3̄*m* phase, the film transitions after 10 minutes to a dual-phase structure with the tetragonal *I*4/*mcm* phase of CsPb_2_X_5_ (indicated by a peak at 2*θ* ∼ 12.17° for the (002) plane) alongside the cubic phase, reflecting a slow halide exchange process. By 20 minutes, the CsPb_2_X_5_ peak intensity decreases, and a new peak at 2*θ* ∼ 31.5° emerges, signifying the orthorhombic *Pnma* phase with increased Cl incorporation, completing the transition at 30 minutes with the splitting of the (002) peak into (004) and (220) planes. This phase evolution, enabled by the core–shell CsPb_2_Br_5_ coating, enhances PQD resistance to UV irradiation and humidity, supporting a LOD of 0.02 ppm for chloride ion detection in tap water as reported.^91^ Panel b shows the fluorescence spectra, with the emission peak shifting and intensifying over the same time intervals, decreasing initially for 20 minutes as the 2D CsPb_2_X_5_ phase forms, then increasing at 30 minutes as it transitions to the CsPbX_3_ orthorhombic *Pnma* phase, indicating a slower halide exchange with NaOCl compared to HCl. Panel C maps these changes in the CIE 1931 color space, with coordinates shifting from (0.0, 0.8) at 0 minutes to (0.3, 0.4) at 30 minutes, offering a visual cue for real-time chloride detection. This stability under UV light and high humidity, facilitated by the core–shell structure, makes the sensor ideal for reliable in-line monitoring in food quality assurance, demonstrating the effectiveness of encapsulation strategies for practical applications in challenging environmental conditions.

Table 6 provides a comprehensive and meticulously curated overview of CsPbBr_3_ PQD-based fluorescence sensors designed for the detection of non-pesticide contaminants, including mycotoxins, heavy metals, illegal dyes, and pharmaceutical residues, reinforcing their pivotal role in advancing food safety diagnostics. It systematically details sensor architectures, such as molecularly imprinted polymer (MIP)-integrated, ratiometric, and ECL-based platforms, achieving LODs as low as 0.027 ng mL^−1^ for PAT and 8.5 fg mL^−1^ for aflatoxin B1. The table elucidates fluorescence modulation mechanisms—PET, FRET, and IFE—underpinning high sensitivity and selectivity in complex food matrices like chili powder, corn oil, and apple juice. Surface engineering strategies, including silica encapsulation, metal–organic framework (MOF) confinement, and ligand functionalization with molecules like 3-mercaptopropionic acid, are highlighted for enhancing aqueous stability and mitigating lead ion leaching, aligning with regulatory requirements.

**Table 6:** Summary of CsPbBr_3_ PQD-based fluorescence sensors for non-pesticide contaminants detection

Contaminant	Detection mechanism	Limit of detection	Sample matrix	Ref.
Sudan I–IV dyes	MIP–PQD	0.3 µg L^−1^	Spices (chilli powder)	50
Rhodamine 6G	Ratiometric FRET	0.01 µg mL^−1^	Water, food	21
Patulin (mycotoxin)	MIP–photopolymer	0.027 ng mL^−1^	Apple juice, jam	22
Aflatoxin B1	ECL (MOF-encapsulated)	3.5 fg mL^−1^	Corn oil, cereals	74
Heavy metals (Cu^2+^)	Ion exchange, PET	0.8 µM	Fruits, tea	76
Tetracycline (antibiotic)	PET (surface engineered)	76 nM	Milk, seafood	26
Ochratoxin A	Smartphone-assisted dual-emission probe	4.1 ng mL^−1^	Coffee, tea, fig, flour	30
2,2-DDVP (organophosphate)	MIP–mesoporous silica	1.27 µg L^−1^	Cabbage, lettuce	72
Prometryn (herbicide)	ECL (MIP–coated QDs)	0.01 µg kg^−1^	Fish, seawater	93
4-Nitroaniline	CsPbBr_3_ PQDs integrated with ZIF-8 MOF	8.4 ppb	Water	94
Rhodamine B	CsPbBr_3_ PQDs integrated with ZIF-8 MOF	0.088 ppm	Water	94
Tetracycline (antibiotic)	CsPbBr_3_/CdS core/shell QDs-based fluorescence sensor	22.6 nM	Water	95
Cu^2+^	All-inorganic CsPbBr_3_ PQDs as photoluminescent probe	0.1 nM	Hexane	96

The unique optical and surface properties of CsPbBr_3_ perovskite QDs have opened new avenues for the detection of a broad range of non-pesticide food contaminants. From banned dyes and antibiotics to heavy metals and mycotoxins, QD-based sensors provide high sensitivity, selectivity, and versatility. Advances in material stability, ratiometric sensing, portable formats, and data integration have moved these technologies closer to practical implementation. However, achieving full regulatory compliance, especially regarding lead safety, and ensuring scalability remain key challenges for future translation from lab to market.

### 5.6. Comparison with other nanoparticles

While CsPbBr_3_ PQDs have demonstrated exceptional promise in fluorescence-based sensing for non-pesticide contaminants, a comparative analysis with established nanomaterials—such as traditional semiconductor quantum dots (*e.g.*, CdSe, InP), metallic nanoparticles (*e.g.*, Au, Ag), carbon dots (CDs), and upconversion nanoparticles (UCNPs)—reveals their unique niche in food safety applications. Unlike CdSe QDs, which suffer from inherent heavy metal toxicity and require complex core–shell architectures to mitigate surface defects, CsPbBr_3_ PQDs offer intrinsically high photoluminescence quantum yields (PLQYs up to 90%) with simpler synthesis routes like hot-injection or LARP.^24,107^ This positions them as a more eco-friendly alternative, albeit with ongoing challenges in aqueous stability those are being addressed through innovative encapsulations. In contrast to Au nanoparticles, which excel in surface plasmon resonance for SERS enhancement but lack intrinsic fluorescence, CsPbBr_3_ PQDs provide direct photoluminescence modulation, enabling label-free assays with minimal instrumentation for contaminants like Sudan dyes.^53^

Optically, CsPbBr_3_ PQDs surpass CDs and UCNPs in spectral purity and brightness; their narrow emission linewidths (<20 nm) minimize overlap with food matrix autofluorescence, unlike the broader spectra of CDs (typically 40–100 nm). This facilitates ratiometric sensing with superior signal-to-noise ratios, as evidenced by LODs as low as 0.027 ng mL^−1^ for patulin, outperforming CD-based sensors that often require additional quenchers or amplifiers. Furthermore, compared to InP QDs, which exhibit lower PLQYs (50–70%) and necessitate toxic precursors, CsPbBr_3_ PQDs achieve tunable bandgap energies through halide exchange, allowing seamless integration into multiplexed platforms for simultaneous detection of mycotoxins and heavy metals without compromising efficiency.^108,111,119^

Stability-wise, CsPbBr_3_ PQDs face competition from robust Ag nanoparticles, which resist environmental degradation but are prone to oxidation in food matrices, leading to inconsistent performance. Encapsulation strategies for CsPbBr_3_, such as MOF or silica hybridization, not only rival the durability of UCNPs (which maintain emission under NIR excitation) but also enhance biocompatibility, reducing non-specific interactions in oily or acidic samples. This adaptability contrasts with the rigidity of metallic nanoparticles, where surface modifications often quench plasmonic effects, limiting their utility in dynamic sensing environments.^72,117^

In terms of sensitivity and selectivity for non-pesticide contaminants, CsPbBr_3_ PQDs integrated with MIPs or aptamers yield sub-nanomolar LODs for tetracycline and heavy metals, eclipsing the micromolar range typical of Au/Ag-based colorimetric assays. Unlike CDs, which rely on aggregation-induced emission but suffer from batch variability, CsPbBr_3_ PQDs support ECL and PEC modalities with enhanced photocurrent stability, as seen in aflatoxin B1 detection at 8.5 fg mL^−1^—orders of magnitude better than traditional QDs in oily matrices.^24,110^ These attributes underscore their versatility in portable formats, bridging the gap between high-end chromatography and field-deployable tools. Ultimately, CsPbBr_3_ PQDs distinguish themselves through a balanced profile of optical excellence, synthetic accessibility, and functional tunability, addressing gaps left by other nanomaterials in regulatory-compliant food sensing. While toxicity mitigation remains a priority, their lead-free variants and hybrid designs promise a transformative edge, fostering innovations that could standardize global food contaminant monitoring with unprecedented precision and ease (Table 7).

**Table 7:** Comparative analysis of CsPbBr_3_ PQDs with other nanomaterials for non-pesticide contaminant sensing in food matrices

Nanomaterial	PLQY (%)	Emission linewidth (nm)	Stability in aqueous matrices	Toxicity concerns	Typical LOD for contaminants (*e.g.*, mycotoxins/heavy metals)	Key advantages in food sensing	Limitations	Ref.
CsPbBr_3_ PQDs	Up to 90	<20	Moderate (improved *via* encapsulation)	Moderate (Pb leaching; lead-free options emerging)	0.027 ng mL^−1^ (patulin); 2 nM (Cu^2+^)	High spectral purity; tunable for multiplexed/ratiometric assays; compatible with ECL/PEC	Aqueous instability; requires surface engineering	24, 53 and 72
CdSe QDs	50–80	20–40	High (core–shell designs)	High (Cd toxicity)	1–10 ng mL^−1^ (mycotoxins); 10–100 nM (metals)	Bright emission; established bioconjugation	Heavy metal risks; complex synthesis	107–109
Au NPs	N/A (plasmonic)	N/A	High	Low	10–100 nM (SERS for dyes)	Plasmon-enhanced SERS; biocompatibility	No intrinsic fluorescence; oxidation-prone	110–112
Carbon dots	10–50	40–100	High	Low	1–10 nM (metals); 0.1–1 µg mL^−1^ (mycotoxins)	Eco-friendly; easy synthesis	Broad emission; batch variability	113–115
UCNPs	0.1–5	<10	High	Low	10–100 fg mL^−1^ (ECL for toxins)	Anti-Stokes emission; deep tissue penetration	Low brightness; requires NIR excitation	116–119
InP QDs	50–70	40–60	Moderate	Moderate (In/P precursors)	0.1–1 µg mL^−1^ (dyes); 50 nM (antibiotics)	Lower toxicity than CdSe; NIR emission options	Lower PLQY; precursor toxicity	120–122

### 5.7. PQD-based detection of pesticides in food samples

PQDs have shown versatility in detecting pesticide residues in food, extending their utility beyond non-pesticide contaminants through fluorescence quenching mechanisms. For isoprothiolane fungicide, CsPbBr_3_ PQDs were synthesized *via* a modified ligand-assisted re-precipitation method using probe sonication, exhibiting strong green fluorescence that undergoes static quenching upon complex formation with isoprothiolane. This enables sensitive detection with a LOD of 10.12 nM and a linear range of 0.025–25 µM, demonstrating excellent selectivity and application to real food samples.^126^ Similarly, for clodinafop pesticide, red-fluorescent CsPbI_3_ PQDs (emission at 686 nm, quantum yield 27%) synthesized by microwave irradiation were integrated with liquid–liquid microextraction (LLME) using hexane, resulting in ∼94% fluorescence turn-off *via* electron transfer quenching. The method achieves an LOD of 34.70 nM, a linear range of 0.1–5 µM, and recoveries of 97–100% with low relative standard deviations in spiked vegetable, fruit, and grain samples.^127^ For pendimethalin pesticide, water-dispersible PS-CsPbBr_3_@CTAB PQDs exhibit bright blue fluorescence (excitation/emission at 370/436 nm) that quenches dramatically upon addition, yielding an LOD of 30.34 nM, a linear range of 5.0–100 µM, good stability, and selective detection with low relative standard deviations in spiked potato and apple samples.^128^

Additional examples further illustrate PQD efficacy: methylammonium lead halide (MAPbX_3_) PQDs enable fluorescence turn-off for polar organochlorine pesticides like methoxychlor through blue shifts in emission spectra, with an LOD of 0.1 ppm and selectivity in agricultural samples.^129^ CsPbBr_3_ PQDs functionalized for chlorpyrifos detection in fruits show quenching-based sensing with an LOD of 0.05 µM and recoveries of 92–105%.^130^ Finally, CsPbCl_3_ PQDs offer turn-off detection for carbendazim fungicide in vegetables, achieving an LOD of 8.5 nM, a linear range of 0.01–10 µM, and high selectivity with recoveries of 95–102%.^76^ These applications highlight the potential of PQDs for pesticide monitoring, though challenges like matrix interference in food samples require ongoing optimization.

### 5.8. Critical assessment of sensing mechanisms, comparative performance with other nanomaterials, and barriers to commercialization

A critical evaluation of the fluorescence modulation mechanisms employed in CsPbBr_3_ PQD-based sensors reveals distinct strengths suited to specific real food matrices. FRET stands out as the most promising for multiplexed detection in complex, autofluorescent samples like milk or apple juice, owing to its high selectivity through proximity-dependent energy transfer and ability to achieve LODs as low as 10 CFU mL^−1^ for pathogens, minimizing background interference.^24,25^ In contrast, PET is highly effective for redox-active non-pesticide contaminants (*e.g.*, heavy metals or antibiotics) in aqueous or semi-solid matrices, leveraging electron transfer for sub-nanomolar sensitivity, but it may be limited by non-specific interactions in lipid-rich foods like oils.^23,46^ AIQ offers simplicity and rapid response for aggregation-prone analytes such as dyes or mycotoxins in homogeneous matrices, yet its susceptibility to false positives from environmental aggregation makes it less ideal for heterogeneous samples.^47^ Ion exchange mechanisms provide unique tunability for ion-specific detection (*e.g.*, Hg^2+^ or Cu^2+^) with spectral shifts, excelling in pH-stable matrices like beverages, but their ionic lattice sensitivity restricts use in acidic or variable-pH foods.^48,51^ Overall, hybrid approaches combining FRET with PET could optimize versatility, balancing selectivity and robustness for diverse food systems.

Comparing CsPbBr_3_ PQDs with other nanomaterials underscores their optical superiority but highlights trade-offs in practical applications. In terms of sensitivity, CsPbBr_3_ PQDs excel with PLQYs up to 90% and narrow emission (<20 nm), enabling LODs of 0.03–3.3 ng mL^−1^ for Sudan dyes *via* PET, outperforming AuNPs' typical micromolar ranges in colorimetric assays due to perovskites' enhanced radiative recombination.^23,107^ Carbon dots (CDs), while offering comparable fluorescence, suffer from broader spectra (40–100 nm), leading to higher spectral overlap and reduced precision in multiplexed sensing for mycotoxins or heavy metals.^113,114^ MOFs provide structural advantages for analyte trapping, achieving similar sub-nanomolar LODs when hybridized with QDs, but alone they lack the intrinsic brightness of perovskites, often requiring additional fluorophores.^72,117^ Thus, CsPbBr_3_ PQDs are particularly advantageous for high-sensitivity fluorescence in trace contaminant detection.

Regarding stability, CsPbBr_3_ PQDs face challenges in aqueous or oxygen-rich food environments, with rapid quenching without encapsulation, unlike AuNPs' robust plasmonic properties that maintain performance in harsh matrices for months.^21,107^ CDs exhibit superior photostability and water dispersibility, retaining fluorescence over extended periods in biological samples, making them more reliable for long-term monitoring without the need for complex coatings.^113,114^ MOFs shine in this aspect, offering hierarchical porosity that protects embedded QDs from degradation, as seen in QD@MOF hybrids retaining 90% PLQY after 140 hours in water—far exceeding bare perovskites.^72,117^ Hybridization with MOFs or silica thus emerges as a key strategy to bridge CsPbBr_3_'s stability gaps, enhancing their viability in real-world food safety.

Regulatory compliance poses a significant differentiator, with CsPbBr_3_ PQDs hindered by Pb^2+^ toxicity and leaching risks conflicting with FDA/WHO limits (<10 ppb in food-contact materials), unlike non-toxic AuNPs and CDs that are more readily approved for direct food applications.^4,113^ AuNPs benefit from established biocompatibility in colorimetric sensors, avoiding heavy metal concerns, while CDs' carbon-based nature aligns with green chemistry standards, facilitating easier integration into regulatory-compliant platforms.^107,114^ MOFs, being tunable and often metal-free in organic frameworks, offer better compliance when used as hosts for perovskites, mitigating toxicity through encapsulation and enabling safer lead-free variants.^72,117^ Addressing Pb issues *via* doping (*e.g.*, Mn^2+^, Bi^3+^) is crucial for perovskites to compete in regulated markets.^19,30^

Practical barriers to commercialization further emphasize the need for targeted improvements in CsPbBr_3_ PQD sensors. Reproducibility remains a challenge, with batch-to-batch variations in size and PLQY (up to 20%) from methods like hot-injection, leading to inconsistent sensor performance and complicating quality control in large-scale production.^28,59^ This contrasts with more uniform synthesis of AuNPs or CDs *via* simpler precipitation routes, which achieve <5% variability and support reliable manufacturing.^107,113^ Toxicity barriers exacerbate commercialization hurdles, as Pb leaching from degraded PQDs violates stringent food safety regulations, necessitating costly lead-free alternatives like CsSnBr_3_ that often compromise optical performance (*e.g.*, 20–30% lower PLQY).^30,97^ While encapsulation reduces risks, it adds processing steps, potentially increasing overall costs by 20–50% and delaying market entry.^21,27^

Synthesis costs represent another key obstacle, with high-temperature hot-injection for CsPbBr_3_ PQDs costing $50–100 g^−1^ due to specialized precursors and equipment, compared to more economical room-temperature LARP at $20–50 g^−1^; however, scaling microfluidics could halve expenses while improving reproducibility.^28,59^ Overcoming these through green, scalable methods and hybrid designs will be essential for translating CsPbBr_3_ PQDs into viable, cost-effective food safety tools. Table 8 provides a critical comparison of CsPbBr_3_ PQDs with AuNPs, carbon dots, and MOFs in key performance metrics for food safety sensing, highlighting perovskites' optical advantages alongside stability and compliance challenges.

**Table 8:** Comparative performance of CsPbBr_3_ PQDs with AuNPs, carbon dots, and MOFs for food safety sensing

Nanomaterial	Sensitivity (*e.g.*, LOD range)	Stability (*e.g.*, in aqueous matrices)	Regulatory compliance	Key ref.
CsPbBr_3_ PQDs	Sub-nanomolar (0.03–3.3 ng mL^−1^ for dyes/mycotoxins)	Moderate; requires encapsulation (*e.g.*, retains 70–90% PLQY up to 140 h)	Limited due to Pb toxicity; needs lead-free variants	21, 30 and 72
AuNPs	Micromolar (1–10 µM for heavy metals/pathogens)	High; resistant to degradation over months	Excellent; non-toxic, FDA-approved for food contact	136
Carbon dots	Nanomolar (1–10 ng mL^−1^ for contaminants)	High; photostable in water/biofluids	Strong; carbon-based, biocompatible	137
MOFs	Sub-nanomolar (when hybridized; 0.1–1 nM for ions)	Excellent; porous structure protects from environmental stress	Good; tunable, often metal-free options	138

## 6. Challenges, prospects, and industrial translation for food safety

While CsPbBr_3_ PQDs offer transformative potential for food safety diagnostics, their practical implementation faces challenges such as environmental instability, lead toxicity, and scalability. This section critically evaluates these barriers, highlights emerging solutions like lead-free perovskites and IoT-integrated platforms, and outlines pathways for industrial translation to enable regulatory-compliant, field-deployable sensing technologies.

### 6.1. Challenges

Despite the promising advancements of CsPbBr_3_ PQDs in fluorescence-based sensing for food safety, a series of practical and scientific challenges must be addressed before these nanomaterials can transition from laboratory prototypes to industrial or consumer-grade diagnostic platforms.

#### 6.1.1. Environmental instability

One of the most persistent obstacles is the intrinsic instability of CsPbBr_3_ PQDs under ambient conditions. These materials are highly sensitive to water, oxygen, light, and heat, leading to phase decomposition, halide migration, and fluorescence quenching. The ionic lattice structure, while beneficial for optoelectronic properties, renders CsPbBr_3_ particularly prone to degradation in aqueous and high-humidity environments—conditions commonly encountered in food samples such as milk, juice, or processed meats.^21,27^ Although encapsulation strategies such as mesoporous silica (SiO_2_) coating, metal–organic framework (MOF) embedding, or polymer shelling have improved QD stability,^30,52^ these methods often complicate the surface chemistry and interfere with target molecule recognition.^139,140^ For instance, silica encapsulation can reduce PLQY by 10–20% due to increased diffusion barriers and trap states, potentially lowering sensitivity.^42^ Additionally, many of these stabilizing layers increase the overall size of the QDs, which can hinder diffusion, reduce signal sensitivity, or compromise the speed of detection.

#### 6.1.2. Lead toxicity

Lead toxicity remains a fundamental barrier to the widespread deployment of CsPbBr_3_ QDs in food diagnostics. The potential for Pb^2+^ leaching from degraded or incompletely encapsulated QDs presents serious health risks and contradicts international food safety regulations. Quantitative studies show leaching levels of 20–50 ppb in unencapsulated PQDs under aqueous stress, exceeding FDA thresholds of 50 ppb for food-contact materials and WHO limits of 10 ppb in drinking water.^22,73^ In encapsulated systems, such as silica-coated PQDs, leaching is reduced to undetectable levels (<10 ppb) even after prolonged immersion in water for over 4 months.^109^ As per WHO and FDA guidelines, the presence of lead in food packaging or sensors is subject to strict limits, especially in devices intended for consumer use. Various strategies—such as metal-ion substitution (*e.g.*, Sn^2+^, Bi^3+^, Mn^2+^) or use of double perovskites (*e.g.*, Cs_2_AgBiBr_6_)—are under exploration to circumvent toxicity concerns. However, these lead-free alternatives still fall short in terms of PLQY (*e.g.*, CsSnBr_3_ ∼50–70% with poor stability due to Sn(ii) oxidation, Cs_2_AgBiBr_6_ ∼0.01–80% with indirect bandgap and low radiative efficiency), structural stability, and synthetic reproducibility compared to CsPbBr_3_ (PLQY up to 90%).^97,98^ Thus, further research is needed to engineer non-toxic perovskite systems with comparable optical performance to CsPbBr_3_.

#### 6.1.3. Surface chemistry and biorecognition

The success of perovskite QD biosensors critically depends on their ability to bind selectively to biological analytes *via* aptamers, antibodies, enzymes, or MIPs. However, post-synthetic surface modification can introduce defect states, induce aggregation, or disrupt colloidal stability—especially when hydrophobic ligands (*e.g.*, oleic acid, oleylamine) are replaced with hydrophilic or bifunctional molecules for aqueous compatibility. In many cases, bioconjugation leads to significant fluorescence quenching, reducing sensor sensitivity.^139,140^ Although encapsulation within inert shells (*e.g.*, SiO_2_ or polymer micelles) can preserve photoluminescence, such strategies often add layers of diffusion resistance that slow down target interaction and reduce signal dynamics.^22,27^ Therefore, optimizing surface chemistry for both biorecognition and optical fidelity remains an unsolved challenge.

#### 6.1.4. Matrix effects and interference

In real-world applications, food matrices contain proteins, fats, carbohydrates, pigments, and metal ions that can interfere with fluorescence signals. Autofluorescence, scattering, and non-specific adsorption frequently generate background noise, reduce signal-to-noise ratio, and lead to false positives or negatives.^29^ These matrix effects are particularly severe in complex samples such as sauces, oils, meats, and fermented products. While MIPs and aptamer-based recognition can improve selectivity, they do not completely eliminate cross-reactivity. Moreover, food processing variables (*e.g.*, temperature, pH, salt concentration) can affect both the QD fluorescence and the stability of the recognition element, making sensor calibration and validation under real-use conditions a major hurdle.

#### 6.1.5. Reproducibility and scalability

Reproducible synthesis of PQDs with consistent size, shape, and optical properties is difficult, especially when scaling from lab-scale synthesis (*e.g.*, hot injection or LARP) to industrial manufacturing. Batch-to-batch variation affects emission peak position, quantum yield, and colloidal stability, ultimately impacting sensing performance and reliability. Microfluidic synthesis and automated continuous-flow reactors have emerged as promising alternatives, offering better control over reaction kinetics and improved uniformity.^28^ However, these technologies are still in early stages for perovskite QDs and require additional development to ensure scalability, cost-effectiveness, and compatibility with surface functionalization steps.

#### 6.1.6. Regulatory and practical barriers

The lack of regulatory validation frameworks, shelf-life studies, and user-centric testing underlines the gap between laboratory research and market readiness. Most CsPbBr_3_ QD-based sensors are tested under controlled conditions with limited sample sizes.^21,24^ Very few studies address real-time monitoring, long-term storage stability, or integration with user-friendly formats such as lateral flow assays or smartphone-readout systems. These limitations must be addressed to gain regulatory approval and commercial acceptance.

### 6.2. Prospects

While the challenges above are non-trivial, the intrinsic optical and structural properties of CsPbBr_3_ QDs make them uniquely suited to revolutionize food safety diagnostics—provided that ongoing innovations are successfully translated into practical systems. One of the most promising directions involves the development of lead-free perovskite analogues that maintain high photoluminescence while eliminating toxicity concerns. CsSnBr_3_, Cs_2_AgBiBr_6_, and Mn^2+^-doped halide perovskites have shown encouraging results with reduced toxicity and enhanced environmental tolerance.^28,30^ However, these alternatives exhibit trade-offs: CsSnBr_3_ achieves PLQY of 50–70% but suffers from rapid Sn(ii) oxidation, leading to poor long-term stability, while Cs_2_AgBiBr_6_ offers PLQY up to 80% in optimized conditions but has an indirect bandgap and low radiative efficiency (*e.g.*, 0.01% at room temperature), limiting its performance compared to CsPbBr_3_ (PLQY up to 90%).^97,98^ In parallel, co-doping strategies—using rare-earth or transition-metal ions such as Mn^2+^, Cu^+^, or Bi^3+^—have been demonstrated to enhance PLQY, stability, and FRET performance, expanding their sensing capabilities while improving safety.^19^ These doped systems also offer tunable emission profiles, supporting multiplexed sensing schemes.

Hybrid QD systems integrated with MOFs, MIPs, and nanocomposites such as POSS (polyhedral oligomeric silsesquioxane) enable simultaneous improvements in stability, selectivity, and signal output. For example, dual-stirring-bar-assisted amplification combined with multicolor perovskite QDs has demonstrated ultralow detection limits for *Salmonella* and *Vibrio* spp.^25^ These advanced composites not only shield the QDs from environmental degradation but also provide hierarchical surface structures for enhanced molecular recognition, though they may introduce minor PLQY reductions (*e.g.*, 5–15% in MOF hybrids due to confinement effects).^73^ The development of biomimetic platforms, such as enzyme-mimicking MIPs or host–guest interactions with cyclodextrins, further expands the range of detectable analytes—from mycotoxins and antibiotics to illegal dyes and preservatives. Additionally, artificial intelligence (AI) integration, including machine learning algorithms like support vector machines (SVM) for signal processing and pattern recognition, can mitigate matrix interferences and achieve classification accuracies up to 98% in multiplexed detection.^29^ AI-driven predictive modeling could further optimize PQD synthesis parameters and sensor calibration, accelerating design cycles and enabling adaptive real-time diagnostics in dynamic food environments.^132^

Ratiometric sensors, which rely on fluorescence intensity ratios rather than absolute values, provide internal referencing that compensates for environmental or instrumental fluctuations. Dual-channel emission systems combining perovskite QDs with carbon dots or doped variants enable high-precision quantitative analysis, even in turbid or colored matrices.^10,99^ This advancement is particularly valuable for visual or smartphone-based diagnostics, where colorimetric transitions can be monitored under UV light and interpreted *via* dedicated apps, reducing reliance on laboratory-grade fluorometers.

The miniaturization of analytical tools through microfluidics and portable photonic systems is rapidly transforming point-of-care food diagnostics. By embedding CsPbBr_3_ QDs into paper microfluidic devices, lateral flow sensors, or smartphone-readout systems, rapid and field-deployable analysis becomes feasible.^52^ Microfluidics also supports high-throughput analysis and low reagent consumption, essential for screening multiple contaminants in parallel. The integration of perovskite QDs into these systems allows for compact, disposable, and low-cost devices that meet the demands of industry, regulators, and consumers alike. Recent trends focus on green chemistry approaches that employ safer solvents, reduce waste, and ensure biocompatibility. For example, aqueous synthesis under mild conditions, ligand engineering with biodegradable surfactants, and solid-state devices are actively being explored to align with environmental and regulatory standards.^67,83^ Moreover, collaborations between academia, industry, and regulatory bodies are essential for establishing quality standards, validation protocols, and product certifications—paving the way for industrial adoption of QD-based food diagnostics.

### 6.3. Industrial translation for food safety applications

The industrial deployment of CsPbBr_3_ PQDs for fluorescence-based sensing of foodborne pathogens and non-pesticide contaminants requires scalable production, efficient integration, and compliance with stringent food safety regulations. Advances in synthesis techniques, such as continuous-flow microfluidics and LARP, enable large-scale production with consistent photoluminescence quantum yields (PLQYs > 85%) and particle size uniformity (<5% deviation).^8,59^ These methods enhance cost-effectiveness compared to traditional techniques like ELISA or HPLC, which are labor-intensive and require centralized facilities.^24^ Automated systems leveraging PQDs can achieve high-throughput screening (50–100 samples per hour), facilitating real-time monitoring in food processing lines for contaminants like *Salmonella* spp. and mycotoxins, aligning with Hazard Analysis and Critical Control Points (HACCP) protocols. Such scalability is pivotal for integrating PQD-based sensors into global food supply chains, particularly in high-volume sectors like dairy and aquaculture.

Stability in complex food matrices remains a critical barrier to commercialization, but recent encapsulation strategies have significantly improved PQD performance. Silica and MOF coatings extend fluorescence stability to over 12 months under ambient conditions, with minimal lead leaching (<10 ppb), meeting FDA and EFSA safety thresholds.^22,73^ For instance, silica-encapsulated PQDs have demonstrated robust detection of aflatoxin B1 in grains with LODs as low as 8.5 fg mL^−1^, outperforming conventional fluorescence assays in oily matrices.^73^ These advancements enable the design of portable, field-deployable devices like paper-based sensors, which offer operational simplicity for on-site testing in resource-limited settings, ensuring compliance with international standards like Codex Alimentarius.^52^

The patent landscape reflects growing innovation in PQD-based food safety sensors, with numerous filings since 2020 focusing on hybrid nanostructures and multiplexed detection platforms.^25,78^ These patents emphasize ratiometric and electrochemiluminescence (ECL) sensors for simultaneous detection of contaminants like tetracycline and Cu^2+^, achieving sub-nanomolar sensitivities in complex matrices such as milk and seafood.^26,77^ Collaborative research under international frameworks, such as the European Food Safety Authority's validation programs, has demonstrated 95% accuracy in real-world trials for *Vibrio parahaemolyticus* detection in aquaculture.^25^ These efforts highlight the potential for integrating PQDs into smart food safety systems, where IoT-enabled devices provide real-time analytics, enhancing traceability and regulatory compliance across global supply chains.^29^

Regulatory challenges, particularly lead toxicity, necessitate the development of lead-free PQD variants like CsSnBr_3_ or Cs_2_AgBiBr_6_, which retain high PLQYs (up to 80%) while eliminating health risks.^30,97^ Recent studies have validated these alternatives for heavy metal detection in vegetables, achieving LODs comparable to CsPbBr_3_ PQDs while adhering to WHO guidelines.^77^ Green chemistry approaches, such as aqueous synthesis with biodegradable ligands, further enhance sustainability, aligning with circular economy principles.^83^ These innovations support the adoption of PQDs in standardized formats like lateral flow assays, which offer practical advantages for small-scale producers in developing regions^24,52^ (Table 9).

**Table 9:** Key innovations and applications of CsPbBr_3_ PQDs in industrial food safety sensing

Innovation	Description	Application in food safety	Development stage	Ref.
Continuous-flow microfluidics	Scalable synthesis using microchannel reactors with precise precursor flow control, achieving PLQY >85% and size uniformity <5%	High-throughput screening of pathogens in seafood	Pilot scale (small-scale industrial trials)	59
Silica/MOF encapsulation	Encapsulation with silica or MOFs, ensuring <10 ppb lead leaching and fluorescence stability >12 months	Mycotoxin detection in grains and oils (LOD 8.5 fg mL^−1^ for aflatoxin B1)	Commercial prototype (validated in complex matrices)	22 and 73
Lead-free PQDs	Sn/Bi-doped variants with PLQY up to 80%, reducing toxicity risks	Heavy metal detection in vegetables (comparable LODs to CsPbBr_3_)	Research phase (laboratory proof-of-concept)	30 and 97
MIP-integrated ratiometric sensors	Molecularly imprinted polymers with PQDs for selective, multiplexed detection	Antibiotic and dye detection in dairy and spices (sub-nanomolar LODs)	Field trials (real-world validation)	25 and 78
IoT-enabled smart sensors	Real-time analytics using machine learning (SVM) with 98% classification accuracy	Traceability in supply chains for pathogen monitoring	Research phase (laboratory-based studies)	29
Paper-based fluorescent sensors	Immobilized PQDs on cellulose for visual detection under UV light	Detection of total polar materials in edible oils (LOD 0.5% w/w)	Commercial prototype (field-deployable)	52

Future industrial translation hinges on establishing robust quality control metrics, such as batch-to-batch reproducibility (<3% PLQY variation) and sensor calibration for matrix effects.^59^ Machine learning-assisted signal processing is emerging as a powerful tool to mitigate autofluorescence and non-specific interactions in food samples, achieving classification accuracies up to 98% for multiplexed pathogen detection.^29^ Continued collaboration between researchers, regulators, and industry stakeholders will be essential to standardize these technologies, ensuring they meet ISO 22000 and other global benchmarks for widespread adoption in food safety monitoring.

## 7. Conclusion

The rising demand for safe, high-quality food has driven the development of rapid, portable sensors for detecting pathogens and contaminants. CsPbBr_3_ PQDs stand out due to their superior photoluminescence, narrow emission, and tunable optical properties. This review explores their synthesis, surface functionalization, and fluorescence-based sensing mechanisms, such as FRET, PET, and AIQ, for detecting foodborne pathogens (*e.g.*, *Salmonella*, *Vibrio*) and contaminants (*e.g.*, mycotoxins, heavy metals). Despite their potential, challenges like environmental instability, lead toxicity, and synthesis reproducibility hinder commercialization. Advances in silica encapsulation, MOF hybridization, and lead-free alternatives aim to overcome these limitations. Future integration with microfluidics, smartphone-based detection, and machine learning could enable field-deployable diagnostics. Ratiometric and multiplexed systems may further enhance accuracy, while green synthesis supports sustainability. By addressing current limitations, CsPbBr_3_ PQDs could revolutionize food safety monitoring, ensuring global food security in the decades ahead.

## Author contributions

Conceptualization: S. I. M., H. S. J., and S. M.; methodology: S. I. M., A. V., and I. B. S.; investigation: S. I. M., H. S. J., R. M. M., S. G., H. Z., R. S., and P. S.; resources: I. B. S., R. M. M., and S. M.; data curation: H. S. J. and A. V.; writing – original draft preparation: S. I. M., H. S. J., A. V., and S. M.; writing – review and editing: S. I. M., H. S. J., A. V., I. B. S., R. M. M., S. G., H. Z., R. S., P. S., and S. M.; visualization: R. M. M. and S. G.; supervision: S. M. and I. B. S.; project administration: S. M.; funding acquisition: S. M. All authors have read and agreed to the published version of the manuscript.

## Conflicts of interest

The authors declare no conflicts of interest.

## Data Availability

No primary research results, software or code have been included and no new data were generated or analysed as part of this review.
